# Global microbial carbonate proliferation after the end-Devonian mass extinction: Mainly controlled by demise of skeletal bioconstructors

**DOI:** 10.1038/srep39694

**Published:** 2016-12-23

**Authors:** Le Yao, Markus Aretz, Jitao Chen, Gregory E. Webb, Xiangdong Wang

**Affiliations:** 1 Key Laboratory of Economic Stratigraphy and Palaeogeography, Nanjing Institute of Geology and Palaeontology, Chinese Academy of Sciences, Nanjing 210008, China; 2 University of Chinese Academy of Sciences, Beijing 100049, China; 3 Université de Toulouse, UPS (OMP), GET, 14 Avenue Edouard Belin, Toulouse F-31400, France; 4 School of Earth Sciences, The University of Queensland, St. Lucia, Queensland 4072, Australia

## Abstract

Microbial carbonates commonly flourished following mass extinction events. The end-Devonian (Hangenberg) mass extinction event is a first-order mass extinction on the scale of the ‘Big Five’ extinctions. However, to date, it is still unclear whether global microbial carbonate proliferation occurred after the Hangenberg event. The earliest known Carboniferous stromatolites on tidal flats are described from intertidal environments of the lowermost Tournaisian (Qianheishan Formation) in northwestern China. With other early Tournaisian microbe-dominated bioconstructions extensively distributed on shelves, the Qianheishan stromatolites support microbial carbonate proliferation after the Hangenberg extinction. Additional support comes from quantitative analysis of the abundance of microbe-dominated bioconstructions through the Famennian and early Tournaisian, which shows that they were globally distributed (between 40° latitude on both sides of the palaeoequator) and that their abundance increased distinctly in the early Tournaisian compared to the latest Devonian (Strunian). Comparison of variations in the relative abundance of skeleton- versus microbe-dominated bioconstructions across the Hangenberg and ‘Big Five’ extinctions suggests that changes in abundance of skeletal bioconstructors may play a first-order control on microbial carbonate proliferation during extinction transitions but that microbial proliferation is not a general necessary feature after mass extinctions.

The Devonian-Carboniferous (D-C) transition was an important interval of biotic and palaeoenvironmental changes, characterized by the end-Devonian Hangenberg mass extinction event^1^,^2^. The Hangenberg mass extinction was a first-order mass extinction on the scale of the ‘Big Five’ extinctions and eliminated >45% genera and ~21% of marine invertebrate families, including many stromatoporoid sponges, corals, ammonoids, conodonts and trilobites^2^,^3^. Recent comprehensive analyses show prolonged and multiple stages during the Hangenberg mass extinction, which resulted from anoxia^1^,^2^,^4^ and climate cooling^1^,^2^ accompanied by prominent changes in sedimentary facies and sea level that lasted from the latest Devonian to the earliest Carboniferous (e.g., ~100–300 kyr; uppermost Lower *Siphonodella praesulcata* Zone to lowest *Siphonodella sulcata* Zone)^2^. The early Tournaisian was a post-extinction interval marked by extensive carbonate deposition driven by relative sea-level rise^2^.

Microbial carbonates flourished globally in the aftermath of the Frasnian-Famennian (F-F) and end-Permian mass extinction events, when resulting metazoan diversity was low^5^. Microbial carbonate resurgence also occurred regionally after the end-Ordovician and end-Triassic extinction events^6^,^7^,^8^. Proliferation of microbial carbonates was generally attributed to reduced competition from multicellular organisms or relaxed ecological constraints after mass extinctions, resulting in lower levels of grazing and/or bioturbation of microbial communities^6^,^9^. In addition to metazoan competition, flourishing microbial carbonates also were supported by a high seawater calcite saturation state (SCSS), which could have enhanced microbial calcification and carbonate production^10^. The SCSS did not obviously change across the D-C transition, remaining as high as in the early Silurian and Famennian and much higher than in the early Triassic^10^. Hence, microbial carbonates should have thrived in the early Tournaisian. Although rare earliest Carboniferous bioconstructions were dominated by stromatolites and/or thrombolites^11^,^12^, to date, it is unclear whether microbial carbonates that formed after the Hangenberg event represent a global microbial post-extinction proliferation or not. In order to test the above hypothesis, we quantitatively reconstructed the variation in abundance of microbial carbonates across the D-C transition.

Although increased microbial carbonate abundance generally was consistent with low metazoan diversity and high SCSS during mass extinction transitions, anomalies were present in some time slices, such as the end-Cretaceous when metazoan diversity declined and SCSS increased, but microbial carbonates did not resurge^10^, possibly as a result of the flourishing calcareous plankton during this time^13^. Furthermore, whether metazoan competition or SCSS dominantly controlled the proliferation of microbial carbonates following the late Silurian extinction event is arguable^14^,^15^. To date, the controlling factors and their potential interactions with microbial carbonate production are complex and still not fully understood in most post-extinction intervals^16^. Secular variations in the Phanerozoic abundance of skeletal reef-building biota and total marine biota^17^, and microbial carbonate and calcified cyanobacteria^10^ are positively correlated, especially during mass extinction transitions. Hence, skeletal and microbial reef ecosystems are important elements of the marine biosphere that could serve as proxies for broader marine metazoan diversity and microbial abundance, respectively. Although the relationship between Phanerozoic metazoan diversity and microbial carbonate abundance has been thoroughly studied^5^,^10^, the relationships between skeletal and microbial bioconstructions have been less well studied. The systematic study of changes in the abundance of skeleton- and microbe-dominated (i.e., bioconstructions formed by microbes, not only those composed of calcimicrobes) bioconstructions across mass extinction transitions could provide new insights into the controlling factors on broader microbial carbonate proliferation.

In this paper, the earliest Carboniferous tidal flat stromatolites after the Hangenberg mass extinction are described briefly from the Qianheishan Formation at the Dashuigou section (GPS: 36°47′17.66″N, 104°56′33.10″E) in Ciyao area, Pingchuan County, central Gansu Province, northwestern China (Fig. 1a,b,c). Then, based on palaeoreef data compiled for this study (Supplementary Table 1), the abundance, composition and distribution of Famennian to early Tournaisian skeleton- and microbe-dominated bioconstructions were quantified, highlighting the evolutionary pattern of bioconstructions during this time interval. Lastly, using the Paleoreefs database of Kiessling *et al*.^18^ and new, recently published data^6^,^7^,^8^,^19^,^20^,^21^,^22^,^23^, the relative abundance of skeleton- and microbe-dominated bioconstructions during the ‘Big Five’ and Hangenberg mass extinction transitions, was systematically reviewed, in order to compare the relationship between skeletal and microbial bioconstructors across the different mass extinction events. Hence, this paper aims to unravel (1) changes in the marine biosphere across the end-Devonian mass extinction transition; and (2) the dominant controlling factor on microbial carbonate proliferation after mass extinctions more generally.

## Results

### Qianheishan stromatolites

**Figure 1:**
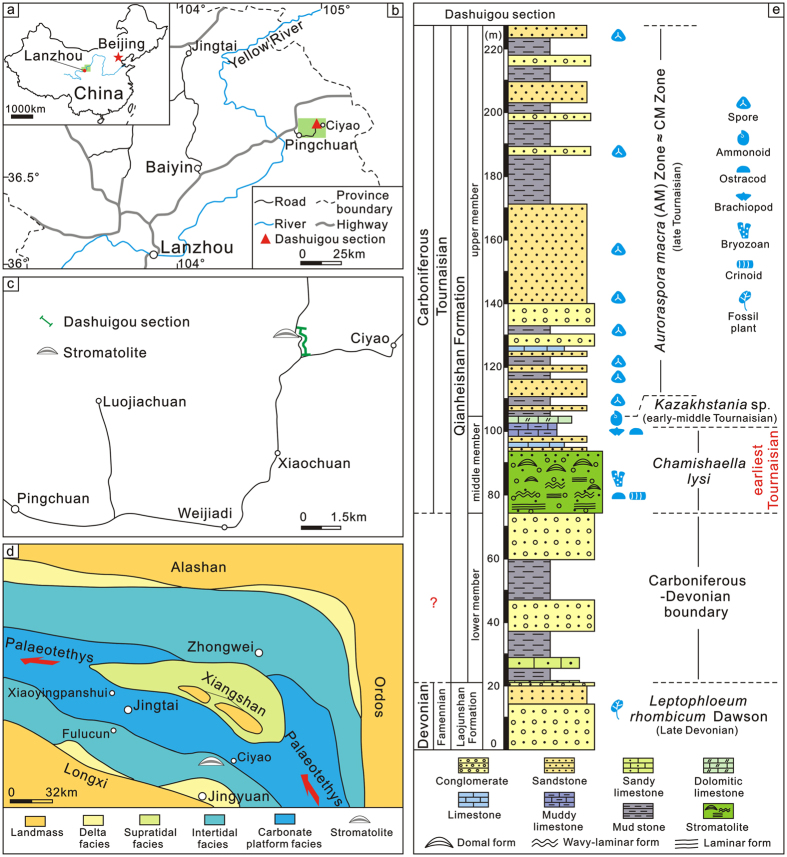
Maps and columnar section. (**a**) Locality map of the capital city (red solid circle) of Gansu Province. (**b**) Amplification of the green shadow area in Fig. 1(a), and the location of the studied section (red triangle) in Pingchuan County, Gansu Province. (**c**) Enlargement of the green shadow area in Fig. 1(b), and the detailed location of the studied section (green solid line) and stromatolites in Ciyao area, Pingchuan County. (**d**) Palaeogeographical map of the studied area and the location of the studied stromatolites. Revised from ref. 24. (**e**) Stratigraphic section showing lithologies and distributions of stromatolites with relevant fossils and their ages. The lithologic column is drawn based on ref. 51 and our field observations. Legends (below) indicate lithologies and stromatolitic morphologies. Symbols (light blue) in the upper right represent fossil occurrences in the studied section or from the equivalent strata in the adjacent area. L.Y. created this figure using CorelDRAW16 (Version number: 16.0.0.843, URL link: http://www.corel.com/cn/).

During the Tournaisian, the Qianheishan stromatolites formed in nearshore facies of the SongPan-GanZi accretionary complex situated between the North and South China Block at ~20° North latitude in the northeastern part of the Palaeotethys Ocean^24^,^25^ (Fig. 1d). In the Dashuigou section, the Qianheishan Formation is the basal unit of Carboniferous strata overlying the late Devonian Laojunshan Formation, which consists mainly of conglomerate and sandstone (Fig. 1e). Consistent with regional stratigraphy, the Qianheishan Formation is divided into three members: a lower member (~52 m) of conglomerate and mudstone with a few nodular limestone beds; a middle member (~33 m) of stromatolitic limestone, bioclastic limestone and dolomitic limestone with a few sandstone and conglomerate beds; and an upper member (~119 m) of mudstone and sandstone (Fig. 1e). Stromatolites occur in the 16 m-thick, lower part of the middle member (Fig. 1e).

The age of the Laojunshan Formation in the Dashuigou section was determined by regional lithostratigraphic correlation with neighboring areas, where the Late Devonian index fossil plant *Leptophloeum rhombicum* Dawson was collected^26^ (Fig. 1e). The occurrence of the ammonoid genus *Kazakhstania* in the upper middle member suggests an early to middle Tournaisian age^27^,^28^ (Fig. 1e). The ostracod genus *Chamishaella*, including *C. aenigmatica, C. brosgei* and *C. lysi*, is abundant in the middle member in the nearby Xiaoyingpanshui section in Jingtai County^29^. The stratigraphic range of *C. lysi* is earliest Tournaisian (former Tnb1)^29^,^30^ (Fig. 1e). Palynological data from the upper member at the Dashuigou section^31^ indicate the *Auroraspora macra* (AM) Zone, which is equivalent to the late Tournaisian *Scbopfites claviger*-*Auroraspora macra* (CM) Zone^32^ (Fig. 1e). Combined, these data support an earliest Tournaisian age for the lower middle member of the Qianheishan Formation, but so far, the D-C boundary has not been precisely determined. In this paper, it is tentatively placed within the lower member (Fig. 1e).

**Figure 2:**
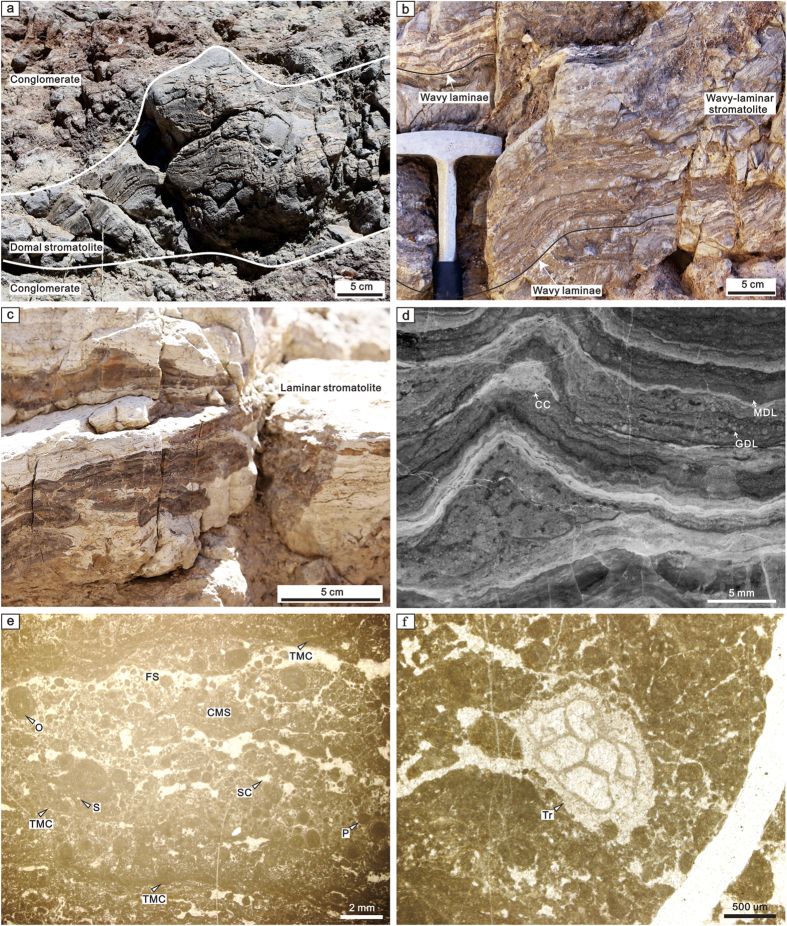
Field, polished-slab and thin-section photographs of the Qianheishan stromatolites at the Dashuigou section, Gansu Province, northwestern China. (**a**) Field photograph of a domal stromatolite intercalated within conglomerate. (**b**) Field photograph of a wavy-laminar stromatolite. (**c**) Field photograph of a laminar stromatolite. (**d**) Polished-slab photograph of a stromatolite with domal, micrite- and grain-dominated laminae and cemented cavity. (**e**) Thin-section photograph of a grain-dominated lamina of the stromatolite. (**f**) Thin-section photograph of the bryozoan from the stromatolite. Abbreviations: CC, Cemented cavity; CMS, Clotted micrite structure; FS, Fenestral structure; GDL, Grain-dominated lamina; MDL, Micrite-dominated lamina; O, Oncoid; P, Peloid; S, Silt; SC, Sparry calcite; TMC, Thin micritic crust; Tr, Trepostome bryozoan.

Qianheishan stromatolites are interbedded with or pass laterally into conglomerate beds (Fig. 2a). The stromatolites contain well-developed light to dark alternating laminae (Fig. 2a,b,c). Three stromatolite morphology types are distinguished, including laminar (Fig. 2c), wavy-laminar (Fig. 2b) and domal forms (Fig. 2a). Domal and wavy-laminar stromatolites may have had depositional relief of ~20 cm and ~5 cm, respectively (Fig. 2a,b). Light and dark laminae are clearly distinguished on polished slabs, containing micrite- and grain-dominated laminae (Fig. 2d). Individual lamina thickness ranges from 1 to 10 mm, and they may pass laterally between different morphological types (Fig. 2d). In microscopic view, laminae contain a variety of components, including micrite, peloids, small oncoids, sparry calcite and silt, together with rare fine to coarse sand-sized detrital grains and bioclasts (e.g., bryozoans) (Fig. 2e,f). Dark, thin micritic crusts were identified between laminae and they in some cases pass gradually into clotted micrite (Fig. 2e). In addition, clotted and fenestral structures occur within laminae (Fig. 2e).

### Famennian-early Tournaisian bioconstruction evolution

**Figure 3:**
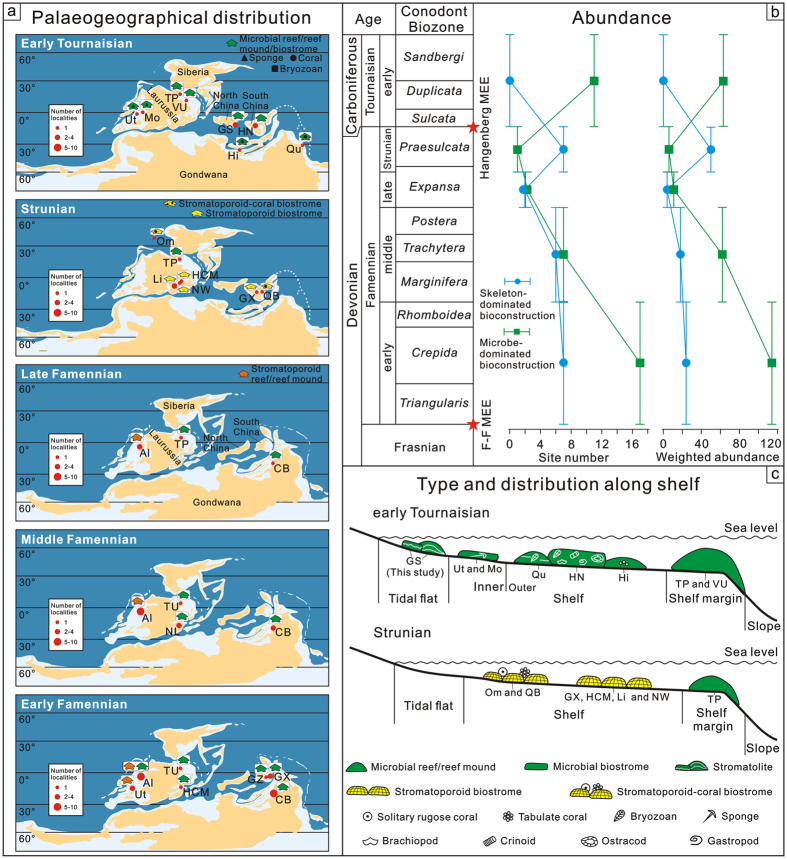
Type, abundance and distribution of the skeleton- and microbe-dominated bioconstructions from the Famennian to the early Tournaisian. (**a**) Composition and global palaeogeographical distribution of the skeleton- and microbe-dominated bioconstructions in the early Tournaisian, Strunian, late Famennian, middle Famennian and early Famennian (with base maps modified from Colorado Plateau Geosystems, Inc. using the Creative Commons Attribution-Share Alike 4.0 International license https://commons.wikimedia.org/wiki/File:340Marect.jpg and 370Marect.jpg). (**b**) Changes in the site number and weighted abundance of skeleton- and microbe-dominated bioconstructions from the Famennian to early Tournaisian. The varied profiles were drawn based on the data from the Supplementary Tables 1 and 2. (**c**) Spatial distribution of skeleton- and microbe-dominated bioconstructions in the early Tournaisian and Strunian. Abbreviations: Al, Alberta, Canada; CB, Canning Basin, Australia; GS, Gansu Province, China; GX, Guangxi Province, China; HCM, Holy Cross Mountains, Poland; Hi, Himalaya, India; Li, Liege, Belgium; MEE, Mass extinction event; Mo, Montana, America; NL, Namur-Liege, Belgium; NW, Northrhine-Westphalier, Germany; Om, Omolon, Russia; QB, Quang Binh, Vietnam; Qu, Queensland, Australia; TP, Timan-Pechora Basin, Russia; TU, Timan-Ural, Russia; Ut, Utah, America; VU, Volga-Ural Province, Russia.

**Figure 4:**
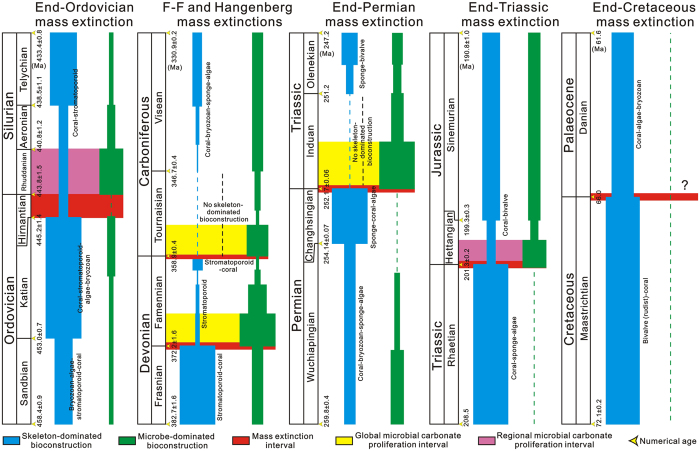
Comparison of the variations in the relative abundance between skeleton- and microbe-dominated bioconstructions across the Hangenberg and ‘Big Five’ mass extinction transitions. Series, stages and numerical ages are according to ref. 52. The ranges of the skeleton- and microbe-dominated bioconstructions are drawn based mainly on the Palaeoreefs database^18^ and this study (Supplementary Tables 1 and 3), and new data for the end-Ordovician transition^6^,^36^, D-C transition^23^,^53^, end-Permian transition^20^,^21^,^38^, end-Triassic transition^7^,^8^,^22^ and end-Cretaceous transition^19^.

In this study, the abundance (site number and weighted abundance), composition and distribution of Famennian to early Tournaisian skeleton- and microbe-dominated bioconstructions were quantitively studied and systematically reviewed for five time slices: early Famennian, middle Famennian, late Famennian, Strunian (latest Famennian) and early Tournaisian (Fig. 3a,b; Supplementary Fig. 1 and Table 1). Famennian to early Tournaisian bioconstructions were widely distributed in Europe, Asia, North America and Australia (Supplementary Fig. 1). In the early Famennian, microbial reefs/reef mounds were very abundant with site number and weighted abundance of 17 and 117 respectively, accompanied by few stromatoporoid reefs with 7 and 24 for site number and weighted abundance, respectively (Fig. 3a,b; Supplementary Table 2). Microbial bioconstructions were globally distributed between 30° latitude on both sides of the palaeoequator, whereas coeval stromatoporoid reefs were restricted to western Laurussia near the palaeoequator (Fig. 3a). Values for site number and weighted abundance of microbial and stromatoporoid reefs/reef mounds decreased gradually from 7 and 62 to 2 and 11 (microbial) and 6 and 18 to 2 and 4 (stromatoporoid) during the middle and late Famennian (Supplementary Table 2), and microbial and stromatoporoid bioconstructions were scarce, being distributed between 30° latitude on both sides of the palaeoequator during this time (Fig. 3a,b). Strunian stromatoporoid-coral biostromes recovered and increased in abundance to site number and weighted abundance of 7 and 50, respectively, and they were located mainly between the palaeoequator and 20° latitude in the southern hemisphere (Fig. 3a,b; Supplementary Table 2). However, microbial reefs were rare during the Strunian with site number and weighted abundance of 1 and 6, respectively (Fig. 3a,b; Supplementary Table 2). To date, no skeleton-dominated bioconstructions have been found in the early Tournaisian (Figs 3 and 4). The site number and weighted abundance of microbial reefs/reef mounds/biostromes distinctly increased with the value of 11 and 63 respectively in the early Tournaisian (Fig. 3b; Supplementary Table 2), which were globally distributed between latitude 40° on both sides of the palaeoequator (Fig. 3a).

### Bioconstruction evolution during ‘Big Five’ transitions

The present paper reviews the relative abundance of skeleton- and microbe-dominated bioconstructions across the ‘Big Five’ mass extinction transitions (Fig. 4). During the Frasnian, carbonate platforms were widely occupied by stromatoporoid-coral bioconstructions with fewer microbe-dominated bioconstructions^33^,^34^ (Fig. 4). The stromatoporoid-coral-microbial reef ecosystem collapsed during the F-F mass extinction, and in the early Famennian microbially dominated reef ecosystems were globally distributed, and few stromatoporoid reefs existed regionally^33^,^35^ (Figs 3 and 4). During the end-Permian mass extinction transition, late Permian (Changhsingian stage) sponge-coral-algal bioconstructions flourished globally with no microbe-dominated bioconstructions, but microbe-dominated bioconstructions proliferated globally in the aftermath of the end-Permian extinction^18^,^21^ (Fig. 4). Skeletal bioconstructions did not recover until the Smithian and then increased in the Spathian when sponge-bivalve bioconstructions occurred^18^,^20^ (Fig. 4). Although similar trends in the abundance of skeleton- and microbe-dominated bioconstructions to those of the F-F and end-Permian extinction transitions occurred during the end-Ordovician and end-Triassic mass extinction intervals, skeleton-dominated bioconstructions were still widely distributed after the end-Ordovician (coral-stromatoporoid) and end-Triassic (coral-bivalve) extinctions^18^,^36^ (Fig. 4). The proliferation of microbial communities and bioconstructions after these two extinctions was regionally restricted to western North America after the end-Ordovician extinction^6^, and to southwestern United Kingdom and southern Sweden after the end-Triassic extinction^7^,^8^ (Fig. 4). At present, no microbe-dominated bioconstructions are known to have occurred during the end-Cretaceous mass extinction transition, and no obvious changes in the abundance of skeleton-dominated bioconstructions have been noted^18^ (Fig. 4).

## Discussion

### Qianheishan stromatolite formation

The interfingering of limestone and conglomerate in the lower part of the middle member of the Qianheishan Formation implies a nearshore, high-energy environment^37^,^38^ (Figs 1e and 2a). The conglomerates may represent increased flux of siliciclastic sediment to a rocky shoreline during shallower parts of parasequences, and the stromatolites were developed farther offshore during transgressive parts of cycles^37^,^38^. This hypothesis is supported by the position of the studied section near a documented palaeolandmass and delta facies^24^ (Fig. 1d). Bryozoans incorporated within the Qianheishan stromatolites (Fig. 2f) suggest that they formed in a marine environment, which was supported by additional reports of bryozoans, crinoids and ostracods in the carbonate facies of the middle member in the Dashuigou section^39^. The fine-scale lamination with silt-sized detritus and fenestral structures in the stromatolites is consistent with an intertidal environment^37^,^38^ (Fig. 2d,e). Additionally, the occurrences of stenohaline biota, such as bryozoans and brachiopods, in the limestones of the middle member of the Qianheishan Formation, indicate that the stromatolites were deposited in a normal marine environment^24^, which is consistent with their locations adjacent to carbonate platform facies and interlinked with Palaeotethys Ocean (Fig. 2d,e).

The Qianheishan stromatolites formed by microbial trapping/binding and *in situ* calcification as they contain both detrital grains and abundant clotted micritic structures, which are widely attributed to calcification of microbial mats^40^ (Fig. 2e). The size of siliciclastic grains (mainly silt) contained in the stromatolites is generally much finer than that of the surrounding sand- and quartz pebbles (Fig. 2a,e), which may result from preferential stabilisation (trapping and binding) of finer mobile grains by microbial mats^41^. Additionally, the fenestrae in the stromatolites may reflect degradation of organic matter by microbes, resulting in open spaces later filled by sparry calcite^42^ (Fig. 2e). The occurrence of steep slopes of the laminae also supports microbial binding in this high energy environment^37^ (Fig. 2a,d).

### Microbial carbonate proliferation

Quantitative analysis of variation in abundance of microbe-dominated bioconstructions across mass extinction transitions allows testing of microbial carbonate proliferation after extinction events. Although Famennian to early Tournaisian bioconstructions have been reviewed globally or regionally^18^,^35^,^43^,^44^,^45^, the age of the bioconstructions is commonly poorly constrained, resulting in reduced knowledge about bioconstruction evolution during this time interval. Hence, it is still unclear if microbial carbonate proliferatied after the end-Devonian mass extinction. In this study, based on the newly constructed pattern of bioconstructions across the boundary (Fig. 3a,b), the site number and weighted abundance of microbe-dominated bioconstructions gradually decreased from the early Famennian to the Strunian (Fig. 3b), as did the palaeogeographical distribution (Fig. 3a). In Strunian times, the site number and weighted abundance of microbe-dominated bioconstructions reached a minimum value, with only rare microbial reefs documented in Russia (Fig. 3a,b). From the Strunian to the early Tournaisian the value of site number and weighted abundance of microbe-dominated bioconstructions increased more than ten-fold (Fig. 3b; Supplementary Table 2), accompanied by increased global distribution between latitude 40° on both sides of the palaeoequator in western America, eastern Russia, eastern Australia, northern India and northwestern and southern China (Fig. 3a; Supplementary Fig. 1 and Table 1). Additionally, microbe-dominated bioconstructions are known in early Tournaisian times across the shelf from the margin and now to onshore tidal flats, which significantly expands their distribution in different ecological zones compared to the Strunian (Fig. 3c). The increased abundance and spatial and ecological expansions suggest improved conditions for microbialite production in the early Tournaisian and thus support global microbial carbonate proliferation after the end-Devonian mass extinction event. The Qianheishan stromatolites provide a new example of microbial proliferation during this time.

### Controlling factors

The Hangenberg mass extinction eliminated >45% genera of marine invertebrates^3^, including dominant reef-building organisms (stromatoporoids and rugose corals), ammonoids, trilobites, conodonts, ostracods, foraminifers, brachiopods, bivalves and some vertebrates (e.g. sharks)^2^. Although some skeletal taxa remained or recovered after the Hangenberg extinction, their diversity levels remained low^2^,^46^,^47^, which could lead to the relaxation of ecological constraints in the early Tournaisian^9^. This hypothesis is supported by the absence of skeleton-dominated bioconstructions during this time (Figs 3b and 4). Some early Tournaisian microbial reefs still contained potential skeletal reef builders (e.g., large colonial rugose corals, syringoporoid corals, bryozoans and solenoporoid algae) but microbialites still dominated by volume (>70%) and stromatolites accounted for more than 30% of the framework^12^. In addition, the early Tournaisian was relatively warm, as evidenced by the scarcity of glacial deposits, transgressive sea levels and low oxygen isotope values compared with the latest Devonian and middle to late Tournaisian^2^,^48^. Elevated early Tournaisian temperatures could have triggered an increase in SCSS^49^, corresponding to increased microbial calcification rates during this time^50^. The occurrences of early Tournaisian microbe-dominated bioconstructions with low abundance of skeletal biota (<5% in volume)^12^ (Fig. 3c), indicate that microbialite-producing biofilms could have competed well against skeletal metazoans under (a) relaxed ecological constraints following the Hangenberg extinction event and (b) increased calcification rates. However, which of the two controlling factors was dominant remains unclear.

Changes in the relative abundance of skeleton- and microbe-dominated bioconstructions, and their comparisons with the “Big Five’ mass extinction transitions, could provide insights into the dominant controlling factor on microbial carbonate proliferation in the aftermath of the mass extinctions. Comparison of the relative abundance of skeleton- and microbe-dominated bioconstructions shows that the latter flourished globally when skeleton-dominated bioconstructions became extinct or declined greatly after mass extinction events, such as the F-F, Hangenberg and end-Permian events (Fig. 4). In contrast, microbe-dominated bioconstructions do not seem to have flourished during mass extinction transitions when there were only small changes in the abundance of skeleton-dominated bioconstructions, as in the end-Cretaceous (Fig. 4). Of course, microbial buildups were not abundant leading up to the end-Cretaceous event either. However, that was also the case leading into the end-Triassic event, yet microbe-dominated buildups flourished at that time (Fig. 4). Microbe-dominated bioconstructions thrived regionally during intervals when the abundance of skeleton-dominated bioconstructions decreased, but they were still distributed widely in the aftermath of mass extinctions, such as the end-Ordovician and end-Triassic (Fig. 4). Hence, the increase in the abundance of microbe-dominated bioconstructions was consistent with the decline of skeleton-dominated bioconstruction abundance after mass extinctions.

Loss of skeleton-dominated bioconstructions after mass extinctions was commonly accompanied by decline in skeletal bioconstructors^17^,^47^, which may have caused the disruption of broader benthic invertebrate communities resulting in the relaxation of ecological constraints in the level-bottom environments and, potentially, low levels of grazing and/or bioturbation of microbial communities^9^. Thus, skeletal bioconstruction ecosystems may have a closer relationship to the ecological constraints that control microbialite producers than does broader metazoan biodiversity itself. Changes in the abundance of skeletal bioconstructors may have provided a first-order control on the proliferation of microbe-dominated bioconstructions. This hypothesis is supported by (1) the mirror-variation trend between metazoan diversity and microbial carbonate abundance^10^, and (2) no positive trend between SCSS and microbial carbonate abundance, although the latter occurred commonly with peak values of SCSS^10^ during the Phanerozoic. However, increased SCSS may have allowed microbial communities to compete better against skeletal constructors during some intervals, such as the early Tournaisian, when potential skeletal bioconstructors were available. Hence, the abundance of skeletal bioconstructors plays a crucial role in regulating the proliferation of microbial carbonates in the aftermath of mass extinctions, but microbial carbonate proliferation is not a necessary feature after mass extinctions.

## Methods

All illustrated specimens are deposited in Nanjing Institute of Geology and Palaeontology, Chinese Academy of Sciences, Nanjing, China. Field photographs were taken using a Canon EOS 6D digital camera. Polished slabs and thin sections were produced using standard techniques, and were photographed with scanner EPSON DS-50000 and microscope Nikon SMZ1500, respectively. The Famennian-early Tournaisian Palaeoreef database in this study was constructed using our own data and published papers on bioconstructions and their biostratigraphy during this time, with inputs from the Paleoreefs database of Kiessling *et al*.^18^. Values of 1, 2, 3 and 4 were assigned to bioconstruction width scales of less than 10 m, 10 to 100 m, 101 to 1000 m and more than 1000 m, respectively, and also to bioconstruction thickness scales of less than 10 m, 10 to 100 m, 101 to 500 m and more than 500 m, respectively. Values of weighted abundance for bioconstruction = (Assumed width value + Assumed thickness value) × Bioconstruction number. The value of site number for bioconstructions represents the number of bioconstruction locations. The database of ‘Big Five’ transitions was created by adding new published data to the extensive data of Kiessling *et al*.^18^ (see Supplementary data, Table 3). The ranges of the relative abundance of bioconstructions across the ‘Big Five’ mass extinction transitions were drawn in accordance with the occurrences of bioconstruction numbers in relevant time slices.

## Additional Information

**How to cite this article:** Yao, L. *et al*. Global microbial carbonate proliferation after the end-Devonian mass extinction: Mainly controlled by demise of skeletal bioconstructors. *Sci. Rep.* **6**, 39694; doi: 10.1038/srep39694 (2016).

**Publisher's note:** Springer Nature remains neutral with regard to jurisdictional claims in published maps and institutional affiliations.

## Supplementary Material

Supplementary Information
