# Supplementary material for: Global microbial carbonate proliferation after the end-Devonian mass extinction: Mainly controlled by demise of skeletal bioconstructors
Source: Sci Rep. 2016 Dec 23;6:39694. doi: 10.1038/srep39694 (PMC5180103; doi:10.1038/srep39694)
Supplement: Supplementary Information [file srep39694-s1.pdf]

Global microbial carbonate proliferation after the end-Devonian mass extinction: Mainly controlled by demise of skeletal bioconstructors

Le Yao[1, 2, 3], Markus Aretz[3*], Jitao Chen[1], Gregory E. Webb[4] & Xiangdong Wang[1*]

[1] Key Laboratory of Economic Stratigraphy and Palaeogeography, Nanjing Institute of Geology and Paleontology, Chinese Academy of Sciences, Nanjing 210008, China.

[2] University of Chinese Academy of Sciences, Beijing 100049, China.

[3] Université de Toulouse, UPS (OMP), GET, 14 Avenue Edouard Belin, Toulouse F-31400, France.

[4] School of Earth Sciences, The University of Queensland, St. Lucia, Queensland 4072, Australia.

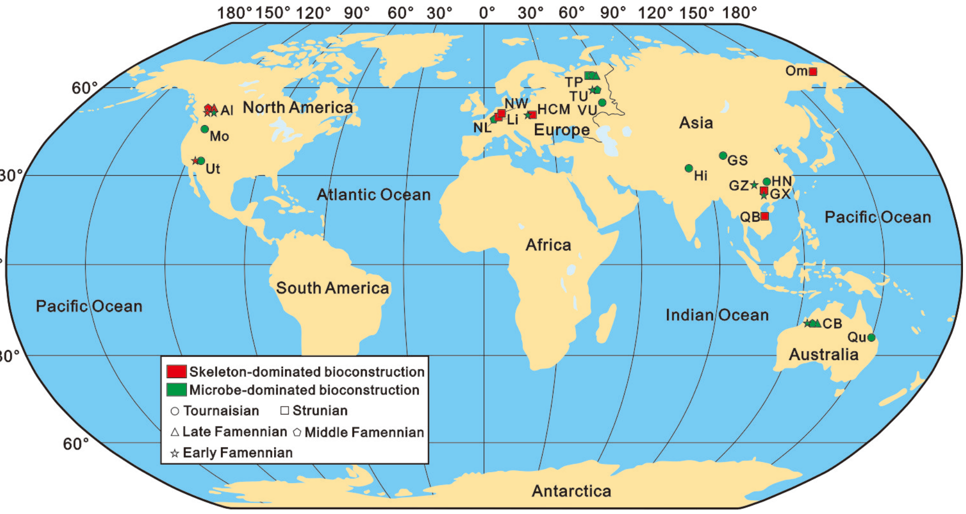

Supplementary figure 1. Location of the Famennian-early Tournaisian skeleton- and microbe-dominated bioconstruction sites on modern global map showing the present-day distribution. The modern global map was modified from Creative Commons with GNU Free Documentation License (https://en.wikipedia.org/wiki/Earth#/media/File:Continents_vide_couleurs.png). Al: Alberta, Canada; CB: Canning Basin, Australia; GS: Gansu Province, China; GX: Guangxi Province, China; HCM: Holy Cross Mountains, Poland; Hi: Himalaya, India; Li: Liege, Belgium; Mo: Montana, America; NL: Namur-Liege, Belgium; NW: Northrhine-Westphalier, Germany; Om: Omolon, Russia; QB: Quang Binh, Vietnam; Qu: Queensland, Australia; TP: Timan-Pechora Basin, Russia; TU: Timan-Ural, Russia; Ut: Utah, America; VU: Volga-Ural Province, Russia. L.Y. created this figure using CorelDRAW16 (Version number: 16.0.0.843, URL link: http://www.corel.com/cn/).

Supplementary table 1. Database of skeleton- and microbe-dominated bioconstructions from the Famennian to early Tournaisian. Width and thickness values of 1, 2, 3 and 4 were assigned for the bioconstruction width scale of less than 10 m, 10 to 100 m, 101 to 1000 m and more than 1000 m, respectively, and for bioconstruction thickness scale of less than 10 m, 10 to 100 m, 101 to 500 m and more than 500 m, respectively. The value of weighted abundance for the bioconstruction is calculated as: (Assumed width value + Assumed thickness value) × Bioconstruction number.

Stage	Reef type	Number	Width	Thickness	Weighted abundance	Bioconstructors	Location	References
Early Tournaisian	Biostrome	1	*10 to 100 m	10 to 100 m	4	Microbes	Ciyao, Pingchuan, Gansu, China	This study
Early Tournaisian	Reef mound	1	*10 to 100 m	10 to 100 m	4	Microbes	Fulu, Jingtai, Gansu, China	Tong, 1996
Early Tournaisian	Biostrome	1	*101 to 1000 m	10 to 100 m	5	Microbes	Malanbian, Xinshao, Hunan, China	Hou et al., 2011
Early Tournaisian	Biostrome	1	*101 to 1000 m	10 to 100 m	5	Microbes	Zhouwangpu, Shaoyang, Hunan, China	Hou et al., 2011
Early Tournaisian	Biostrome	1	*10 to 100 m	Less than 10 m	3	Microbes	Qiyang, Sujiaping, Hunan, China	Hou et al., 2011
Early Tournaisian	True reef	3	10 to 100 m	Less than 10 m	9	Microbes (Main), Bryozoans (Secondary)	Gudman, Rockhampton, Queensland, Australia	Webb, 1998, 2005
Early Tournaisian	True reef	1	More than 1000 m	101-500 m	7	Microbes	Volga-Ural Province, Russia	Antoshkina, 1998; Ulmishek, 1988
Early Tournaisian	True reef	1	More than 1000 m	101-500 m	7	Microbes	Timan-Pechora Basin, Russia	Antoshkina, 1998; Ulmishek, 1988
Early Tournaisian	Reef mound	>1 (*2)	*Less than 10 m	Less than 10 m	4	Microbes (Main), Tabulate corals	Himalaya, India	Bhargava, 1997; Draganits et al.,

						(Secondary)		2002
Early Tournaisian	Biostrome	2	*10-100 m	Less than 10 m	6	Microbes (Main), Siliceous sponges (Secondary)	Utah, America	Gutschick and Perry, 1959
Early Tournaisian	Biostrome	3	*10-100 m	Less than 10 m	9	Microbes (Main), Siliceous sponges (Secondary)	Southwestern Montana, America	Gutschick and Perry, 1959
Strunian	True reef	1	More than 1000 m	10 to 100 m	6	Microbes	Timan-Pechora Basin, Russia	Ulmishek, 1988
Strunian	Biostrome	1	10-100 m	10-100 m	4	Stromatoporoids	Dison, Liege, Belgium	Aretz and Chevalier, 2007
Strunian	Biostrome	3	10-100 m	Less than 10 m (2), 10-100m (1)	10	Stromatoporoids	Dolhain, Liege, Belgium	Aretz and Chevalier, 2007
Strunian	Biostrome	1	*10-100 m	*Less than 10 m	3	Stromatoporoids (Main), Corals (Secondary)	Phong Nha, Quang Binh, Vietnam	Nguyen and Mistiaen, 1998
Strunian	Biostrome	7	10-100 m	Less than 10 m	21	Stromatoporoids	Guilin, Guangxi, China	Kiessling et al., 2003; Milhau et al., 1997
Strunian	Biostrome	1	More than 1000 m	10-100 m	6	Stromatoporoids	Holy Cross Mountains, Krakow, Poland	Berkowski, 2002; Kiessling et al., 2003
Strunian	Biostrome	1	10-100 m	Less than 10 m	3	Stromatoporoids	Northrhine-Westphalia, Germany	Herbig and Weber, 1996
Strunian	Biostrome	1	*10-100 m	Less than 10 m	3	Corals (Main), Stromatoporoids	Pushok, Omolon, Russia	Kiessling et al., 2003; Simakov et

						(Secondary)		al., 1983
Late Famennian	True reef	1	*Less than 10 m	Less than 10 m	2	Stromatoporoids	Jura Creek, Alberta, Canada	Peterhänsel and Pratt, 2008
Late Famennian	True reef	1	*Less than 10 m	Less than 10 m	2	Stromatoporoids	Southesk River, Alberta, Canada	Peterhänsel and Pratt, 2008
Late Famennian	True reef	1	More than 1000 m	101-500 m	7	Microbes	Timan-Pechora Basin, Russia	Ulmishek, 1988
Late Famennian	True reef/ Reef Mound	2	Less than 10 m	Less than 10 m	4	Microbes	Dingo Cap, Napier Range, Canning Basin, Australia	George et al., 1997
Middle Famennian	True reef	1	101-1000 m	Less than 10 m	4	Stromatoporoids (Main), Microbes (Secondary)	Normandville, Alberta, Canada	Stearn, 1988; Halim-Dihardja and Mountjoy, 1989
Middle Famennian	True reef	1	*10-100 m	10-100 m	4	Stromatoporoids	Hart Range, Alberta, Canada	Peterhänsel and Pratt, 2008
Middle Famennian	True reef	1	*10-100 m	10-100 m	4	Stromatoporoids	Nigel Peak, Alberta, Canada	Peterhänsel and Pratt, 2008
Middle Famennian	True reef	1	*Less than 10 m	Less than 10 m	2	Stromatoporoids	Hummingbird Creek, Alberta, Canada	Peterhänsel and Pratt, 2008
Middle Famennian	True reef	1	*Less than 10 m	Less than 10 m	2	Stromatoporoids	Jura Creek, Alberta, Canada	Peterhänsel and Pratt, 2008
Middle Famennian	True reef	1	*Less than 10 m	Less than 10 m	2	Stromatoporoids	Southesk River, Alberta, Canada	Peterhänsel and Pratt, 2008
Middle Famennian	True reef	5	10-100 m	Less than 10 m	15	Microbes	Baelen, Liege, Belgium	Aretz and Chevalier, 2007
Middle	True reef	1	10-100 m	Less than 10	3	Microbes	Baelen, Liege, Belgium	Aretz and Chevalier,

Famennian				m				2007
Middle Famennian	True reef	1	10-100 m	Less than 10 m	3	Microbes	Lesse Valley, Namur, Belgium	Aretz and Chevalier, 2007
Middle Famennian	True reef/ Reef Mound	12	Less than 10 m	Less than 10 m	24	Microbes	Dingo Cap, Napier Range, Canning Basin, Australia	George et al., 1997
Middle Famennian	True reef/ Reef Mound	3	Less than 10 m	Less than 10 m	6	Microbes	McSHERRY'S Cap, Napier Range, Canning Basin, Australia	George et al., 1997
Middle Famennian	True reef/ Reef Mound	2	Less than 10 m	Less than 10 m	4	Microbes	Barker River, Napier Range, Canning Basin, Australia	George and Chow, 2002
Middle Famennian	Reef Mound	1	More than 1000 m	101-1000 m	7	Microbes	Chernyshev Swell, Timan-northern Ural, Russia	Antoshkina, 2006
Early Famennian	True reef	2	*Less than 10 m	Less than 10 m	4	Stromatoporoids (Main), Microbes (Secondary)	Jasper, Alberta, Canada	Pratt and Peterhansel, 1997
Early Famennian	True reef	1	*10-100 m	10-100 m	4	Stromatoporoids	Normandville, Alberta, Canada	Peterhänsel and Pratt, 2008
Early Famennian	True reef	1	*10-100 m	10-100 m	4	Stromatoporoids	Hummingbird Creek, Alberta, Canada	Peterhänsel and Pratt, 2008
Early Famennian	True reef	1	*Less than 10 m	Less than 10 m	2	Stromatoporoids	Crowsnest Pass, Alberta, Canada	Peterhänsel and Pratt, 2008
Early Famennian	True reef	1	*Less than 10 m	Less than 10 m	2	Stromatoporoids	Overturn Mountain, Alberta, Canada	Peterhänsel and Pratt, 2008
Early	True reef	1	*10-100 m	10-100 m	4	Stromatoporoids	Crossfield, Alberta,	Peterhänsel and

Famennian							Canada	Pratt, 2008
Early Famennian	True reef	1	*10-100 m	10-100 m	4	Stromatoporoids	Bullion Canyon, northern Dugway Range, westcentral Utah, America	Morrow et al., 2011
Early Famennian	True reef	1	*10-100 m	10-100 m	4	Stromatoporoids	Buckhorn Canyon, northern Dugway Range, westcentral Utah, America	Morrow et al., 2011
Early Famennian	True reef	1	10-100 m	10-100 m	4	Microbes	Zhaijiang, Guangxi, China	Shen et al., 1997
Early Famennian	True reef	1	10-100 m	10-100 m	4	Microbes	Miaomen, Guangxi, China	Shen and Webb, 2004a
Early Famennian	True reef	1	101-1000 m	10-100 m	5	Microbes	Shatang, Guangxi, China	Shen and Webb, 2004b
Early Famennian	Biostrome	1	More than 1000 m	Less than 10 m	5	Microbes	Maoying, Guizhou, China	Shen et al., 2006
Early Famennian	True reef	2	Less than 10 m (1), 10-100 m (1)	Less than 10 m	5	Microbes (Main), Sponges (Secondary)	Windjana Gorge, Napier Range, Canning Basin, Australia	Wood, R., 2000
Early Famennian	True reef	3	Less than 10 m	Less than 10 m	6	Microbes	Chedda Cliffs, Canning Basin, Australia	Chow and George, 2004
Early Famennian	True reef/Reef mound	7	*Less than 10 m	Less than 10 m	14	Microbes	NW Oscar Range, Canning Basin, Australia	Stephens and Sumner, 2003
Early	True	6	Less than 10	Less than 10	12	Microbes	Dingo Cap, Napier	George et al., 1997

Famennian	reef/Reef mound		m	m			Range, Canning Basin, Australia	
Early Famennian	True reef/Reef mound	8	Less than 10 m	Less than 10 m	16	Microbes	Barker River, Napier Range, Canning Basin, Australia	George and Chow, 2002
Early Famennian	True reef/Reef mound	4	Less than 10 m	Less than 10 m	8	Microbes	Carpenter Gap, Napier Range, Canning Basin, Australia	George and Chow, 2002
Early Famennian	True reef/Reef mound	3	Less than 10 m	Less than 10 m	6	Microbes	East Windjana Gorge, Napier Range, Canning Basin, Australia	George and Chow, 2002
Early Famennian	True reef/Reef mound	2	Less than 10 m	Less than 10 m	4	Microbes	NRD-3, Napier Range, Canning Basin, Australia	George and Chow, 2002
Early Famennian	Reef mound	5	Less than 10 m	Less than 10 m	10	Microbes (Main), Stromatoporoids (Secondary)	Northeastern Horse Spring Range, Canning Basin, Australia	Webb, 2001
Early Famennian	Reef mound	1	10-100 m	10-100 m	4	Microbes (Main), Stromatoporoids (Secondary)	Southwestern Horseshoe Range, Canning Basin, Australia	Webb, 2001
Early Famennian	True reef/Reef mound	1	Less than 10 m	Less than 10 m	2	Microbes	Psie Górki, Holy Cross Mountains, Poland	Rakociński and Racki, 2016
Early Famennian	Reef mound	3	Less than 10 m	Less than 10 m	6	Microbes	Ancient Wall, Alberta, Canada	Whalen et al., 2002

Early Famennian	Reef mound	1	More than 1000 m	10-100 m	6	Microbes	Chernyshev Swell, Timan-northern Ural, Russia	Antoshkina, 2006

* For assumed values, for true reef/reef mound the assumed value of their width is the same as their thickness value. For biostromes, the assumed value of their width is one grade larger than their thickness value.

Supplementary table 2. Site numbers and weighted abundance of skeleton- and microbe-dominated bioconstructions for the early Tournaisian, Strunian, late Famennian, middle Famennian and early Famennian.

Stage	Site number		Weighted abundance	
	Skeleton-dominated bioconstruction	Microbe-dominated bioconstruction	Skeleton-dominated bioconstruction	Microbe-dominated bioconstruction
Early Tournaisian	0	11	0	63
Strunian	7	1	50	6
Late Famennian	2	2	4	11
Middle Famennian	6	7	18	62
Early Famennian	7	17	24	117

Supplementary table 3. Database of the skeleton- and microbe-dominated bioconstructions from the “Big Five” mass extinction transitions and early Carboniferous.

Stage	Reef type	Number	Bioconstructors	Location	References
Late Ordovician					
Sandbian	True reef	2	Bryozoans	Tennessee and Alabama, America	Webby, 2002; Kiessling et al., 2003
Sandbian	True reef	3	Bryozoans	Virginia and Tennessee, America	Webby, 2002; Kiessling et al., 2003
Sandbian	True reef	5	Bryozoans	South central Oklahoma, America	Webby, 2002; Kiessling et al., 2003

Sandbian	True reef	1	Corals	Newfoundland, America	Webby, 2002; Kiessling et al., 2003
Sandbian	True reef	1	Corals, stromatoporoids, algae	Tennessee and Alabama, America	Webby, 2002; Kiessling et al., 2003
Sandbian	True reef	1	Corals, stromatoporoids, algae	Newfoundland, America	Webby, 2002; Kiessling et al., 2003
Sandbian	True reef	1	Corals, stromatoporoids, algae	Tiewadian, Shanxi, China	Webby, 2002; Kiessling et al., 2003
Sandbian	True reef	2	stromatoporoids	Tiewadian, Shanxi, China	Webby, 2002; Kiessling et al., 2003
Sandbian	True reef	1	algae	Abralinski Synclinorium, Kazakhstan	Webby, 2002; Kiessling et al., 2003
Sandbian	True reef	1	algae	Lianglitag, Bachu, China	Webby, 2002; Kiessling et al., 2003
Sandbian	True reef	1	algae	Tianshan area, China	Webby, 2002; Kiessling et al., 2003
Sandbian	True reef	1	algae	Tiewadian, Shanxi, China	Webby, 2002; Kiessling et al., 2003
Sandbian	True reef	1	microbes	S.Ont., America	Webby, 2002; Kiessling et al., 2003
Katian	True reef	1	Corals, Stromatoporoids	S.Quebec, America	Webby, 2002; Kiessling et al., 2003
Katian	True reef	3	Corals, Stromatoporoids	South Norway	Webby, 2002; Kiessling et al., 2003
Katian	True reef	3	Corals, Stromatoporoids	Dongzhuang, Shanxi, China	Webby, 2002; Kiessling et al., 2003
Katian	True reef	1	Corals, Stromatoporoids	Qingling, S. Shanxi, China	Webby, 2002; Kiessling et al., 2003
Katian	True reef	1	Corals, stromatoporoids,	Boonderoo, Cliefden Caves, New South Wales, Australia	Webby, 2002; Kiessling et al., 2003

			algae		
Katian	True reef	1	Corals, stromatoporoids, algae	South Norway	Webby, 2002; Kiessling et al., 2003
Katian	True reef	3	Stromatoporoids	South Norway	Webby, 2002; Kiessling et al., 2003
Katian	True reef	1	Stromatoporoids	Boonderoo, Cliefden Caves, New South Wales, Australia	Webby, 2002; Kiessling et al., 2003
Katian	True reef	1	Corals	N. Qilian area, China	Webby, 2002; Kiessling et al., 2003
Katian	True reef	1	Corals	Jiaboscar area, China	Webby, 2002; Kiessling et al., 2003
Katian	True reef	1	Corals	Boonderoo, Cliefden Caves, New South Wales, Australia	Webby, 2002; Kiessling et al., 2003
Katian	True reef	1	Corals	Gunning bland, New South Wales, Australia	Webby, 2002; Kiessling et al., 2003
Katian	True reef	1	Corals	Central Norway	Webby, 2002; Kiessling et al., 2003
Katian	True reef	1	Corals	Estonia	Webby, 2002; Kiessling et al., 2003
Katian	True reef	3	Corals	N. Urals, Russia	Webby, 2002; Kiessling et al., 2003
Katian	True reef	1	Bryozoans	S .Quebec, America	Webby, 2002; Kiessling et al., 2003
Katian	True reef	1	Bryozoans	Estonia	Webby, 2002; Kiessling et al., 2003
Katian	True reef	1	Bryozoans	Jiaboscar area, China	Webby, 2002; Kiessling et al., 2003
Katian	True reef	3	Algae	South Norway	Webby, 2002; Kiessling et al., 2003
Katian	True reef	1	Algae	Vaigach, Russia	Webby, 2002; Kiessling et al., 2003
Katian	True reef	1	Algae	Ishim River, Kazakhstan	Webby, 2002; Kiessling et al., 2003
Katian	True reef	1	Algae	Maltisor atoll, Kazakhstan	Webby, 2002; Kiessling et al., 2003
Katian	True reef	4	Algae	Anderken Range, Kazakhstan	Webby, 2002; Kiessling et al., 2003
Katian	True reef	1	Algae	Maikain area, Kazakhstan	Webby, 2002; Kiessling et al., 2003
Katian	True reef	1	Algae	Karakol River, Kyrgyzstan	Webby, 2002; Kiessling et al., 2003

Katian	True reef	1	Algae	Dongzhuang, Shanxi, China	Webby, 2002; Kiessling et al., 2003
Katian	True reef	1	Algae	Qinglin area, China	Webby, 2002; Kiessling et al., 2003
Katian	True reef	1	Microbes	Dongzhuang, Shanxi, China	Webby, 2002; Kiessling et al., 2003
Katian	True reef	2	Corals	Anzhar River, Kazakhstan	Webby, 2002; Kiessling et al., 2003
Katian	True reef	1	Corals	West Mongolia	Webby, 2002; Kiessling et al., 2003
Katian	True reef	1	Corals	Vaigach, Russia	Webby, 2002; Kiessling et al., 2003
Katian	True reef	2	Corals	N. Urals, Russia	Webby, 2002; Kiessling et al., 2003
Katian	True reef	1	Corals	Morkoka River, Russia	Webby, 2002; Kiessling et al., 2003
Katian	True reef	2	Corals	Sette-Daban and Verkhoyanranges, Russia	Webby, 2002; Kiessling et al., 2003
Katian	True reef	3	Corals	Elgenchak Mts, Russia	Webby, 2002; Kiessling et al., 2003
Katian	True reef	1	Corals	Omulev Mts, Russia	Webby, 2002; Kiessling et al., 2003
Katian	True reef	1	Corals	Penzhina Range and Koryakh foothills, Russia	
Katian	True reef	1	Bryozoans	N. Urals, Russia	Webby, 2002; Kiessling et al., 2003
Katian	True reef	1	Bryozoans	S.Ontario, America	Webby, 2002; Kiessling et al., 2003
Katian	True reef	1	Bryozoans	Kentucky, America	Webby, 2002; Kiessling et al., 2003
Katian	True reef	1	Corals, Stromatoporoids	Mackenzie Mts, Canada	Webby, 2002; Kiessling et al., 2003
Katian	True reef	1	Corals, Stromatoporoids	Paykhoi, Russia	Webby, 2002; Kiessling et al., 2003
Katian	True reef	1	Corals, Stromatoporoids	Taymyr Peninsula, Russia	Webby, 2002; Kiessling et al., 2003
Katian	True reef	1	Corals, Stromatoporoids	Podkamennaya Tunguska River basin, Russia	Webby, 2002; Kiessling et al., 2003
Katian	True reef	1	Corals,	Sette-Daban and Verkhoyanranges,	Webby, 2002; Kiessling et al., 2003

			Stromatoporoids	Russia	
Katian	True reef	1	Corals, Stromatoporoids	Tarim Basin, China	Webby, 2002; Kiessling et al., 2003
Katian	True reef	1	Corals, Stromatoporoids	N. Qilian, China	Webby, 2002; Kiessling et al., 2003
Katian	True reef	1	Corals, Stromatoporoids	Qaidamu Basin, China	Webby, 2002; Kiessling et al., 2003
Katian	True reef	2	Corals, stromatoporoids, algae	Yushan, Jiangxi, China	Webby, 2002; Kiessling et al., 2003
Katian	True reef	2	Corals, stromatoporoids, algae	Tas-Khayakhtakh Range, Russia	Webby, 2002; Kiessling et al., 2003
Katian	True reef	3	Corals, stromatoporoids, algae	Rassokha and Serechen rivers	Webby, 2002; Kiessling et al., 2003
Katian	True reef	3	Corals, stromatoporoids, algae	Chukoiskil Peninsula, Russia	Webby, 2002; Kiessling et al., 2003
Katian	True reef	2	Alage	N. Urals, Russia	Webby, 2002; Kiessling et al., 2003
Katian	True reef	1	Alage	Gomy Altai, Russia	Webby, 2002; Kiessling et al., 2003
Katian	True reef	1	Microbes	Southampton Island, Canada	Webby, 2002; Kiessling et al., 2003
Katian	True reef	1	Microbes	Melville Peninsula, Canada	Webby, 2002; Kiessling et al., 2003
Hirnantian	True reef	1	Corals	Anticosti Island, America	Webby, 2002; Kiessling et al., 2003
Hirnantian	True reef	1	Corals	Sette-Daban and Verkhoyanranges, Russia	Webby, 2002; Kiessling et al., 2003

Hirnantian	True reef	1	Corals	Penzhina Range and Koryakh foothills, Russia	Webby, 2002; Kiessling et al., 2003
Hirnantian	True reef	2	Corals, stromatoporoids, algae	Tas-Khayakhtakh Range, Russia	Webby, 2002; Kiessling et al., 2003
Hirnantian	True reef	2	Corals, stromatoporoids	South Norway	Webby, 2002; Kiessling et al., 2003
Hirnantian	True reef	1	Corals, stromatoporoids	N. Urals, Russia	Webby, 2002; Kiessling et al., 2003
Hirnantian	True reef	2	algae	N. Urals, Russia	Webby, 2002; Kiessling et al., 2003
			Early Silurian		
Rhuddanian	True reef	1	Stromatoporoids, corals	Hilleste, Hiiumaa Island, Estonia	Kiessling et al., 2003
Rhuddanian	True reef	1	stromatoporoids, Corals	Rockville, Manitoulin Isl., Ontario, Canada	Kiessling et al., 2003
Rhuddanian	True reef	1	Corals	Melville Peninsula, N.W.T., Canada	Kiessling et al., 2003
Rhuddanian	True reef	1	Corals, Stromatoporoids	Manitowaning, Manitoulin Isl., Ontario, Canada	Kiessling et al., 2003
Rhuddanian	True reef	1	Corals, Stromatoporoids	Pusku-Suurekivi, Ridala Peninsula, Estonia	Kiessling et al., 2003
Rhuddanian	Reef mound	1	Bryozoans	Honora Bay, Manitoulin Isl., Ontario, Canada	Kiessling et al., 2003
Rhuddanian	Reef mound	1	Bryozoans	Drummond Island II, Michigan, United States	Kiessling et al., 2003
Rhuddanian	Reef mound	1	Corals	Rigby Bay, Devon Is., N.W.T., Canada	Kiessling et al., 2003
Rhuddanian	Reef mound	1	Stromatoporoids	Bessels Fjord II, Greenland, Denmark	Kiessling et al., 2003

Rhuddanian	Reef mound	>18	Microbes	Nevada and Utah, United States	Sheehan and Harris, 2004
Aeronian	True reef	1	Corals, stromatoporoids	Cross Lake SE, Manitoba, Canada	Kiessling et al., 2003
Aeronian	True reef	1	Corals, stromatoporoids	Baerum, Norway	Kiessling et al., 2003
Aeronian	True reef	1	Corals, stromatoporoids	Reed North Quarry, Fairborn, Green Co., Ohio, United States	Kiessling et al., 2003
Aeronian	True reef	1	Corals, stromatoporoids	Shiqian, Guizhou, China	Kiessling et al., 2003
Aeronian	True reef	1	Corals, stromatoporoids	Diebu, S Gansu, China	Kiessling et al., 2003
Aeronian	True reef	1	Stromatoporoids, corals	Eastern Ellesmere Island, Canada South Wales, Australia	Kiessling et al., 2003
Aeronian	Reef mound	1	Corals	East Point, Anticosti Isl., Quebec, Canada	Kiessling et al., 2003
Aeronian	Reef mound	1	Stromatoporoids, corals	Mansel Island, N.W.T., Canada	Kiessling et al., 2003
Aeronian	Biostrome	1	Corals, stromatoporoids	Grand Rapids, Manitoba, Canada	Kiessling et al., 2003
Aeronian	True reef	1	Microbes	Fenggnang, Guizhou, China	Kiessling et al., 2003
Aeronian	Biostrome	1	Microbes	Bijie area, NW Guizhou, China	Kiessling et al., 2003
Telychian	True reef	1	Stromatoporoids, corals	Akimiski Island, Hudson Bay, Nunavut, Canada	Kiessling et al., 2003
Telychian	True reef	1	Stromatoporoids, corals	Pen Island No. 1, Hudson Bay, Ontario, Canada	Kiessling et al., 2003
Telychian	True reef	1	Stromatoporoids,	Gavu Fm., Mushgai, Mongolia	Kiessling et al., 2003

			corals		
Telychian	True reef	1	Stromatoporoids, corals	Tompo and Vost Khandygi Rivers, Russia	Kiessling et al., 2003
Telychian	True reef	1	Stromatoporoids, corals	Severn River, Ontario, Canada	Kiessling et al., 2003
Telychian	True reef	1	Stromatoporoids, corals	Cap River, Anticosti Isl., Quebec, Canada	Kiessling et al., 2003
Telychian	True reef	1	Stromatoporoids, bryozoans	Attawapiskat River, Ontario, Canada	Kiessling et al., 2003
Telychian	True reef	1	Corals	Cheng'kou II, Sichuan, China	Kiessling et al., 2003
Telychian	True reef	1	Corals	Wangjiawan, Ningqiang County, Shaanxi, China	Kiessling et al., 2003
Telychian	True reef	1	Corals, stromatoporoids	Sharchuluut Fm., Shine-Jinst, Mongolia	Kiessling et al., 2003
Telychian	True reef	1	Corals, stromatoporoids	Molong High, New South Wales, Australia	Kiessling et al., 2003
Telychian	True reef	1	Corals, stromatoporoids	Fort Island, Cedar Lake, Manitoba, Canada	Kiessling et al., 2003
Telychian	True reef	1	Corals, bryozoans	Elwood quarry, Clinton Co., Iowa, United States	Kiessling et al., 2003
Telychian	True reef	1	Microbes	Lec-Elec River, Pechora, Russia	Kiessling et al., 2003
Telychian	Reef mound	1	Stromatoporoids, corals	Comeault No. 1, Hudson Bay, Ontario, Canada	Kiessling et al., 2003
Telychian	Reef mound	1	Stromatoporoids, corals	Börglum Elv, Peary Land, Greenland, Denmark	Kiessling et al., 2003
Telychian	Reef mound	1	Stromatoporoids,	Valdemar Glückstadt Land, Greenland,	Kiessling et al., 2003

			corals	Denmark	
Telychian	Reef mound	1	Stromatoporoids, corals	Kap Independence, Washington Land, Greenland, Denmark	Kiessling et al., 2003
Telychian	Reef mound	1	Stromatoporoids	Prince Alfred Bay, Devon Is., N.W.T., Canada	Kiessling et al., 2003
Telychian	Reef mound	1	Corals, stromatoporoids	Huashito, Sichuan, China	Kiessling et al., 2003
Telychian	Reef mound	1	Corals, stromatoporoids	Severnaya Zemlya, Srednij bioherms, Russia	Kiessling et al., 2003
Telychian	Reef mound	1	Corals, stromatoporoids	SW Point, Anticosti Isl., Quebec, Canada	Kiessling et al., 2003
Telychian	Reef mound	1	Corals, stromatoporoids	Khalyya River, Sette Daban Mountains, Russia	Kiessling et al., 2003
Telychian	Reef mound	1	Corals	Visby, Gotland, Sweden	Kiessling et al., 2003
Telychian	Reef mound	1	Bryozoans, Corals	Gascons, Gaspé, Quebec, Canada	Kiessling et al., 2003
Telychian	Biostrome	1	Corals	Harricana River, Quebec, Canada	Kiessling et al., 2003
Telychian	Biostrome	1	Corals, stromatoporoids	Dashiyan, Sichuan, China	Kiessling et al., 2003
Telychian	Biostrome	1	Corals, stromatoporoids	Lake Agassiz, Manitoba, Canada	Kiessling et al., 2003
Telychian	Biostrome	1	Corals, stromatoporoids	Fairford, Cedar Lake, Manitoba, Canada	Kiessling et al., 2003
Telychian	Biostrome	1	Corals, stromatoporoids	Moose Lake Settlement, Manitoba, Canada	Kiessling et al., 2003
Telychian	Biostrome	1	Stromatoporoids, corals	Attawapiskat River, Ontario, Canada	Kiessling et al., 2003

			Late Devonian		
Early Frasnian	True reef	1	Corals, stromatoporoids	Kemerovo, Kuznesk Basin, Siberia, Russia	Kiessling et al., 2003
Early Frasnian	True reef	1	Stromatoporoids, corals	Fort Good Hope, N.W.T., Canada	Kiessling et al., 2003
Early Frasnian	Reef mound	1	Corals, algae	Guppy Hills, Canning Basin, Australia	Kiessling et al., 2003
Early Frasnian	Biostrome	1	Stromatoporoids, corals	Ferques, Boulonnais, France Russia	Kiessling et al., 2003
Middle Frasnian	True reef	1	Stromatoporoids, microbes	Kotovo, Volgograd, Russia	Kiessling et al., 2003
Middle Frasnian	True reef	1	Stromatoporoids, microbes	Golden Spike, Alberta, Canada	Kiessling et al., 2003
Middle Frasnian	True reef	1	Stromatoporoids, corals	Dyminy reef, Grabina, Kielce, Holy Cross Mts., Poland	Kiessling et al., 2003
Middle Frasnian	True reef	1	Stromatoporoids, corals	Oused Marinelli, Carnic Alps, Italy	Kiessling et al., 2003
Middle Frasnian	True reef	1	Stromatoporoids, corals	Mercy Bay, Banks Island, N.W.T., Canada	Kiessling et al., 2003
Middle Frasnian	True reef	1	Stromatoporoids, corals	Heart Lake, Great Slave Lake, N.W.T., Canada	Kiessling et al., 2003
Middle Frasnian	True reef	1	Corals, stromatoporoids	Alexandra, Hay River, N.W.T., Canada	Kiessling et al., 2003
Middle Frasnian	True reef	1	Microbes, stromatoporoids	Tarasovo, Volgograd, Russia	Kiessling et al., 2003
Middle	Reef mound	1	Stromatoporoids,	Barvaux, Belgium	Kiessling et al., 2003

Frasnian			corals		
Middle Frasnian	Reef mound	1	Stromatoporoids, corals	Hummingbird and Whiterabbit, Alberta, Canada	Kiessling et al., 2003
Middle Frasnian	Reef mound	1	Corals, stromatoporoids	Layavozh, Pechora, Russia	Kiessling et al., 2003
Middle Frasnian	Reef mound	1	Corals, stromatoporoids	Voyvozh, Timan, Russia	Kiessling et al., 2003
Middle Frasnian	Reef mound	1	Microbes, stromatoporoids	Limonovka, central Urals, Russia	Kiessling et al., 2003
Upper Frasnian	True reef	1	Stromatoporoids, corals	Glubokaya River, Kuznesk Basin, Siberia, Russia	Kiessling et al., 2003
Upper Frasnian	True reef	1	Corals, algae	W Pembina Field, Alberta, Canada	Kiessling et al., 2003
Upper Frasnian	True reef	1	Stromatoporoids, microbes	July Lake area, British Columbia, Canada	Kiessling et al., 2003
Upper Frasnian	True reef	1	Stromatoporoids	Arrowhead, Jean Marie, N.W.T., Canada	Kiessling et al., 2003
Upper Frasnian	Reef mound	1	Corals, stromatoporoids	W Tebuk, Timan, Russia	Kiessling et al., 2003
Upper Frasnian	Reef mound	1	Corals, stromatoporoids	Challis, Lost River Range, Custer Co., Idaho, United States	Kiessling et al., 2003
Upper Frasnian	Reef mound	1	Corals, stromatoporoids	Nisku, Bigoray, Alberta, Canada	Kiessling et al., 2003
Upper Frasnian	Reef mound	1	Corals, stromatoporoids	Whitehorse, Jasper Basin, Alberta, Canada	Kiessling et al., 2003
Upper	Reef mound	1	Stromatoporoids,	Shar'yu River section, Chernyshev	Kiessling et al., 2003

Frasnian			microbes	Ridge, Russia	
Upper Frasnian	Reef mound	1	Calcisponges, corals	Ice Fall Brook Canyon, B. C., Canada	Kiessling et al., 2003
Upper Frasnian	Reef mound	1	Corals, Stromatoporoids	Inya River, Russia	Kiessling et al., 2003
Upper Frasnian	Reef mound	1	Corals, Stromatoporoids	Celibeta, Jean Marie, N.W.T., Canada	Kiessling et al., 2003
Upper Frasnian	Reef mound	1	Stromatoporoids, corals	Middle Kakisa River, N.W.T., Canada	Kiessling et al., 2003
Upper Frasnian	Reef mound	1	Microbes	Mukhanovo-Yerokhovo depression, Russia	Kiessling et al., 2003
Upper Frasnian	Reef mound	1	Microbes, corals	Rocky Pass, Jasper Basin, Alberta, Canada	Kiessling et al., 2003
Upper Frasnian	Biostrome	1	Stromatoporoids, corals	Dingo Gap, Napier Range, Canning Basin, Australia	Kiessling et al., 2003
Upper Frasnian	Biostrome	1	Stromatoporoids, corals	Mokrá, Moravia, Czechia	Kiessling et al., 2003
Upper Frasnian	Biostrome	1	Stromatoporoids, corals	Coral Falls, Trout River, Canada	Kiessling et al., 2003
Upper Frasnian	Biostrome	1	Stromatoporoids, corals	Foetus Lake, N.W.T., Canada	Kiessling et al., 2003
Upper Frasnian	Biostrome	1	Stromatoporoids	Central Iowa, United States	Kiessling et al., 2003
Upper Frasnian	Biostrome	1	Stromatoporoids	Deep Lake, N.W.T., Canada	Kiessling et al., 2003
Upper	Biostrome	1	Corals,	Carlson Lake, N.W.T., Canada	Kiessling et al., 2003

Frasnian			stromatoporoids		
Upper Frasnian	Biostrome	1	Corals, stromatoporoids	Tathlina Lake, N.W.T., Canada	Kiessling et al., 2003
Upper Frasnian	Biostrome	1	Corals, stromatoporoids	Kakisa Lake, N.W.T., Canada	Kiessling et al., 2003
Frasnian	True reef	1	Stromatoporoids, corals	McWhae Ridge, Canning Basin, Australia	Kiessling et al., 2003
Frasnian	True reef	1	Stromatoporoids, corals	Windjana Gorge, Canning Basin, Australia	Kiessling et al., 2003
Frasnian	True reef	1	Stromatoporoids, corals	Langenaubach-Breitscheid, Germany	Kiessling et al., 2003
Frasnian	True reef	1	Stromatoporoids, corals	Unknown Locality, Latvia	Kiessling et al., 2003
Frasnian	True reef	1	Stromatoporoids, corals	Grosmont, Alberta, Canada	Kiessling et al., 2003
Frasnian	True reef	1	Stromatoporoids, corals	Grosmont II, Alberta, Canada	Kiessling et al., 2003
Frasnian	True reef	1	Stromatoporoids, corals	Flathead Range, Alberta, Canada	Kiessling et al., 2003
Frasnian	True reef	1	Stromatoporoids, corals	Buzuluk depression, Russia	Kiessling et al., 2003
Frasnian	True reef	1	Stromatoporoids, corals	Clearwater River, Fairholme, Alberta, Canada	Kiessling et al., 2003
Frasnian	True reef	1	Stromatoporoids, corals	Cripple Creek, Fairholme, Alberta, Canada	Kiessling et al., 2003
Frasnian	True reef	1	Stromatoporoids	Sturgeon Lake, Alberta, Canada	Kiessling et al., 2003

Frasnian	True reef	1	Stromatoporoids	Cirrus Mountain, Southesk Cairn, Alberta, Canada	Kiessling et al., 2003
Frasnian	True reef	1	Stromatoporoids, microbes	Teplov, Russia	Kiessling et al., 2003
Frasnian	True reef	1	Stromatoporoids, algae	Swan Hills Buildup, Alberta, Canada	Kiessling et al., 2003
Frasnian	True reef	1	Corals	Corandos Island, Prince of Wales Island, Alaska, United States	Kiessling et al., 2003
Frasnian	True reef	1	Corals	S Verkhoyansk, Russia	Kiessling et al., 2003
Frasnian	True reef	1	Corals	Kondoma River, Kuznezsk, Russia	Kiessling et al., 2003
Frasnian	True reef	1	Corals, stromatoporoids	Vaygach Island, Russia	Kiessling et al., 2003
Frasnian	True reef	1	Corals, stromatoporoids	Uralo-Povolzhiya, Russia	Kiessling et al., 2003
Frasnian	True reef	1	Corals, stromatoporoids	Vitebsk, Belarus	Kiessling et al., 2003
Frasnian	True reef	1	Corals, stromatoporoids	Pripyat Depression, Belarus	Kiessling et al., 2003
Frasnian	True reef	1	Corals, stromatoporoids	Forbes Creek, Banff, Alberta, Canada	Kiessling et al., 2003
Frasnian	True reef	1	Microbes	Yasachnoy River, B. Tuonnakh, Russia	Kiessling et al., 2003
Frasnian	True reef	1	Microbes	North Kharyakha, Pechora, Russia	Kiessling et al., 2003
Frasnian	True reef	1	Microbes, stromatoporoids	Vuktyl-Dzhebol depression E, Russia	Kiessling et al., 2003
Frasnian	Reef mound	1	Stromatoporoids, corals	Karawanken, Austria	Kiessling et al., 2003

Frasnian	Reef mound	1	Stromatoporoids, corals	Saratov region, Russia	Kiessling et al., 2003
Frasnian	Reef mound	1	Stromatoporoids, corals	Tas-Khayakhtakh Range II, Russia	Kiessling et al., 2003
Frasnian	Reef mound	1	Stromatoporoids, corals	Vermilion Falls, Peace River, Alberta, Canada	Kiessling et al., 2003
Frasnian	Reef mound	1	Stromatoporoids, corals	Leduc & Acheson, Alberta, Canada	Kiessling et al., 2003
Frasnian	Reef mound	1	Stromatoporoids, corals	Ancient Wall, Alberta, Canada	Kiessling et al., 2003
Frasnian	Reef mound	1	Stromatoporoids, corals	Hranice, Moravia, Czechia	Kiessling et al., 2003
Frasnian	Reef mound	1	Stromatoporoids, algae	Burnt Timber, Fairholme, Alberta, Canada	Kiessling et al., 2003
Frasnian	Reef mound	1	Stromatoporoids	Koh-e Zardak, Afghanistan	Kiessling et al., 2003
Frasnian	Reef mound	1	Stromatoporoids	Inzer River, Urals, Russia	Kiessling et al., 2003
Frasnian	Reef mound	1	Stromatoporoids	Chosuov River, Urals, Russia	Kiessling et al., 2003
Frasnian	Reef mound	1	Stromatoporoids	Bonnie Glen, Alberta, Canada	Kiessling et al., 2003
Frasnian	Reef mound	1	Corals, stromatoporoids	Gamburtsev Uplift, Pechora, Russia	Kiessling et al., 2003
Frasnian	Reef mound	1	Corals	Bokan West, Afghanistan	
Frasnian	Reef mound	1	Corals	Kolyvan-Tomsk Trough IV, Russia	Kiessling et al., 2003
Frasnian	Reef mound	1	Corals	Mt. McDougall, Fairholme, Alberta, Canada	Kiessling et al., 2003
Frasnian	Reef mound	1	Corals	Allstones Creek, Alberta, Canada	Kiessling et al., 2003
Frasnian	Reef mound	1	Bryozoans, corals,	Mount Sayed Tabib, Afghanistan	Kiessling et al., 2003

Frasnian	Reef mound	1	Serpulids, worms, microbes	Yavapai Co., Arizona, United States	Kiessling et al., 2003
Frasnian	Reef mound	1	Microbes	South Pashnya, Timan, Russia	Kiessling et al., 2003
Frasnian	Reef mound	1	Microbes	Yuryakha, Pechora, Russia	Kiessling et al., 2003
Frasnian	Reef mound	1	Microbes, stromatoporoids	Unnamed, Pechora, Russia	Kiessling et al., 2003
Frasnian	Reef mound	1	Microbes, corals	Mount Irish, Lincoln Co., Nevada, United States	Kiessling et al., 2003
Frasnian	Biostrome	1	Stromatoporoids, corals	Bidu River, Kerman, Iran	Kiessling et al., 2003
Frasnian	Biostrome	1	Stromatoporoids, corals	Mountain Springs, Clark Co., Nevada, United States	Kiessling et al., 2003
Frasnian	Biostrome	1	Stromatoporoids, corals	Safi, Morocco	Kiessling et al., 2003
Frasnian	Biostrome	1	Stromatoporoids, corals	Shell Rock River, Iowa, United States	Kiessling et al., 2003
Frasnian	Biostrome	1	Stromatoporoids, microbes	White Man Gap, Canmore, Alberta, Canada	Kiessling et al., 2003
Frasnian	Biostrome	1	Stromatoporoids	Ghuk, Afghanistan	Kiessling et al., 2003
Frasnian	Biostrome	1	Corals, stromatoporoids	Table Rock Rapids, Trout River, Canada	Kiessling et al., 2003
Early Carboniferous					
Middle Tournaisian	Reef mound	1	Microbes	Chukoiskil Peninsula, Russia	Aretz and Webb, 2006
Late Tournaisian	Biostrome	2	Microbes	Nova Scotia, southeastern Canada	Gallacher, 2010; Burg, 2013

Early Visean	True reef	1	Corals	Marton Quarry, Furness, England, United Kingdom	Kiessling et al., 2003
Early Visean	True reef	1	Corals, bryozoans	Weaver Hills District, Staffordshire, England, United Kingdom	Kiessling et al., 2003
Early Visean	True reef	1	Microbes, algae	Popeshall Quarry Buildup, Dublin, Ireland	Kiessling et al., 2003
Early Visean	Biostrome	1	Microbes, bryozoans	Hadnock, Wales, Great Britain, United Kingdom	Kiessling et al., 2003
Early Visean	Biostrome	1	Microbes, corals	Stack Cleugh, Bewcastle, England, United Kingdom	Kiessling et al., 2003
Early Visean	Biostrome	1	Microbes, algae	Clonalvy Buildup, Dublin, Ireland	Kiessling et al., 2003
Middle Visean	True reef	1	Microbes, bryozoans	Namur, Belgium	Kiessling et al., 2003
Middle Visean	Reef mound	1	Microbes, corals	Salmon Hill Buildup, Dublin, Ireland	Kiessling et al., 2003
Middle Visean	Reef mound	1	Corals, bryozoans	Engihoul Quarry, Liege, Belgium	Kiessling et al., 2003
Middle Visean	Biostrome	1	Corals	Yashui, Guizhou, China	Yao et al., 2016
Middle Visean	Biostrome	4	Corals	Engihoul, Corhalie, Bomel and Polderlee, Belgium	Yao et al., 2016
Middle Visean	Biostrome	1	Microbes	Yatton, Bristol, England, United Kingdom	Kiessling et al., 2003
Late Visean	True reef	1	Corals, bryozoans	Akiyoshi Terrane, southern Japan	Yao et al., 2016
Late Visean	True reef	1	Corals	Tiouinine, near Khenifra, central Morocco	Yao et al., 2016
Late Visean	True reef	1	Corals, algae	Rockhampton, Queensland, Australia	Yao et al., 2016
Late Visean	True reef	1	Corals	Gower Peninsula, southern Wales, UK	Yao et al., 2016
Late Visean	True reef	1	Corals	Castelsec, Montagne Noire, France	Yao et al., 2016
Late Visean	True reef	1	Corals, bryozoans	Rockhampton, Queensland, Australia	Yao et al., 2016

Late Visean	True reef	1	Microbial, sponge, bryozoan, coral	Hadim region, Taurides, S Turkey	Yao et al., 2016
Late Visean	True reef	1	Microbes, sponges	Jerada Massif, NE Morocco	Yao et al., 2016
Late Visean	Reef mound	1	Bryozoans	Llandudno, North Wales, UK	Yao et al., 2016
Late Visean	Reef mound	1	Bryozoans, microbes	Port au Port Peninsula, western Newfoundland, Canada	Yao et al., 2016
Late Visean	Reef mound	1	Microbes	Eastern Sahara, Algeria	Kiessling et al., 2003
Late Visean	Reef mound	>2	Microbes	Laibin, Guangxi, South China	Yao and Wang, 2016
Late Visean	Reef mound	1	Microbes, bryozoans	Charlestown, Scotland, United Kingdom	Kiessling et al., 2003
Late Visean	Reef mound	1	Microbes, sponges, bryozoans, corals	Stebden Hill, North Yorkshire, northern England	Mundy, 1994
Late Visean	Biostrome	2	Corals	Royseux, Southeast Belgium	Yao et al., 2016
Late Visean	Biostrome	2	Corals	Southeastern and northwestern Ireland	Yao et al., 2016
Late Visean	Biostrome	1	Corals	Sierra Morena area, southwestern Spain	Yao et al., 2016
Late Visean	Biostrome	1	Corals, sponges	Little Asby Scar, northern England	Yao et al., 2016
Late Visean	Biostrome	1	Corals	Adarouch, central Morroco	Yao et al., 2016
Visean	True reef	1	Microbes, corals	Werris Creek, N.S.W., Australia	Kiessling et al., 2003
			Late Permian		
Wuchiapingian	True reef	1	Microbes, bryozoans	Unknown	Kiessling et al., 2003
Wuchiapingian	True reef	1	Microbes, bryozoans	Unknown	Kiessling et al., 2003
Wuchiapingian	Reef mound	1	Bryozoans, microbes	Aberford, England, United Kingdom	Kiessling et al., 2003
Wuchiapingian	Reef mound	1	Bryozoans, microbes	Maltby, England, United Kingdom	Kiessling et al., 2003
Wuchiapingian	Reef mound	1	Corals, calcisponges	Djare Sebak, Khoja Murod, Herirod, Afghanistan	Kiessling et al., 2003

Wuchiapingian	Reef mound	1	Algae, tubiphytes	Nikitinskaya Gorge, Caucasus, Russia	Kiessling et al., 2003
Wuchiapingian	Reef mound	1	Tubiphytes, Calcisponges	Argolis, Greece	Kiessling et al., 2003
Wuchiapingian	Reef mound	1	Microbes, bryozoans	E-Durham, England, United Kingdom	Kiessling et al., 2003
Wuchiapingian	Reef mound	1	Microbes, bryozoans	Pössneck, Germany	Kiessling et al., 2003
Wuchiapingian	Reef mound	1	Microbes, bryozoans	Römerstein, Harz, Germany	Kiessling et al., 2003
Wuchiapingian	Reef mound	1	Microbes, bryozoans	Schale, Germany	Kiessling et al., 2003
Wuchiapingian	Reef mound	1	Microbes, bryozoans	Abenra, Denmark	Kiessling et al., 2003
Wuchiapingian	Biostrome	1	Corals	Qincaiyuan, Ziyun, Guizhou, China	Kiessling et al., 2003
Wuchiapingian	Biostrome	1	Corals, tubiphytes	Hanlong, Guizhou, China	Kiessling et al., 2003
Changhsingian	True reef	1	Calcisponges, stromatoporoids	Jianshuigou Reefs, Chuenmuping, Sichuan, China	Kiessling et al., 2003
Changhsingian	True reef	1	Calcisponges, stromatoporoids	Jiantianba, Hubei, China	Kiessling et al., 2003
Changhsingian	True reef	1	Calcisponges, stromatoporoids	Unknown	Kiessling et al., 2003
Changhsingian	True reef	1	Calcisponges, stromatoporoids	Unknown	Kiessling et al., 2003
Changhsingian	True reef	1	Calcisponges, bryozoans	Ziyun County, Guizhou, China	Kiessling et al., 2003
Changhsingian	True reef	1	Calcisponges, bryozoans	Unknown	Kiessling et al., 2003
Changhsingian	True reef	1	Calcisponges, algae	Unknown	Kiessling et al., 2003
Changhsingian	True reef	1	Calcisponges, algae	Daluokeng, Gaofeng, Cili Co.,	Kiessling et al., 2003

				Hunan, China	
Changhsingian	True reef	1	Calcisponges, microbes	Unknown	Kiessling et al., 2003
Changhsingian	True reef	1	Corals, calcisponges	Kangjia-ping, Cili, Hunan, China	Kiessling et al., 2003
Changhsingian	True reef	1	Algae, stromatoporoids	Wangmo, Guizhou, China	Kiessling et al., 2003
Changhsingian	Reef mound	1	Calcisponges	Hydra, Greece	Kiessling et al., 2003
Changhsingian	Reef mound	1	Calcisponges, stromatoporoids	Huangnitang, Hubei, China	Kiessling et al., 2003
Changhsingian	Reef mound	1	Calcisponges, bryozoans	Tudiya, Beipei buildup group, Sichuan, China	Kiessling et al., 2003
Changhsingian	Biostrome	1	Calcisponges, Siliceous sponges	Phrae, Thailand	Kiessling et al., 2003
Changhsingian	Biostrome	1	Calcisponges, microbes	Skyros, Greece	Kiessling et al., 2003
Changhsingian	Biostrome	1	Corals	Zigui, Hubei, China	Kiessling et al., 2003
Early Triassic					
Griesbachian	Biostrome	1	Microbes	Unknown	Kiessling et al., 2003
Griesbachian	Biostrome	1	Microbes	northern Pakistan	Mata and Bottjer, 2012
Griesbachian	Biostrome	1	Microbes	Curuk Dag, Turkey	Mata and Bottjer, 2012
Griesbachian	Biostrome	>3	Microbes	Sichuang, Chongqi and Hubei, South China	Mata and Bottjer, 2012
Griesbachian	Biostrome	1	Microbes	Bulla parastratotype, Italy	Mata and Bottjer, 2012
Griesbachian	Biostrome	1	Microbes	Hungary	Mata and Bottjer, 2012
Griesbachian	Biostrome	1	Microbes	Southwest Japan	Mata and Bottjer, 2012
Griesbachian	Biostrome	>1	Microbes	Guizhou, South China	Mata and Bottjer, 2012
Griesbachian-Dienerian	Biostrome	1	Microbes	Oman	Mata and Bottjer, 2012
Griesbachian-	Biostrome	1	Microbes	northern Hungary	Mata and Bottjer, 2012

Smithian					
Dienerian-Smithian	Biostrome	1	Microbes	Oman	Mata and Bottjer, 2012
Dienerian-Spathian	Biostrome	1	Microbes	northern Iraq	Mata and Bottjer, 2012
Smithian	Reef mound	>3	Sponges	Utah, United States	Brayard et al., 2011
Smithian-Spathian	Biostrome	1	Microbes	Guizhou, China	Mata and Bottjer, 2012
Spathian	Reef mound	>4	Bivalves	Nevada and Utah, United States	Brayard et al., 2011
Smithian	Reef mound	>5	Sponges	Nevada and Utah, United States	Brayard et al., 2011
Spathian	Biostrome	1	Microbes	Nevada, USA	Mata and Bottjer, 2012
Spathian	Biostrome	1	Microbes	Iran	Mata and Bottjer, 2012
Induan	Reef mound	1	Microbes	Unknown	Kiessling et al., 2003
Induan	Reef mound	1	Microbes	Unknown	Kiessling et al., 2003
			Late Triassic		
Rhaetian	True reef	1	Corals	Apuseni Mts., Romania	Kiessling et al., 2003
Rhaetian	True reef	1	Corals	Fischerwiese, Steinplatte, Austria	Kiessling et al., 2003
Rhaetian	True reef	1	Corals, calcisponges	Wilde Kirche and Tegernsee, Austria	Kiessling et al., 2003
Rhaetian	True reef	1	Corals, calcisponges	Adnet, Salzburg, Austria	Kiessling et al., 2003
Rhaetian	True reef	1	Corals, calcisponges	Malajon, Palawan, Philippines	Kiessling et al., 2003
Rhaetian	True reef	1	Corals, alage	Korfu, Greece	Kiessling et al., 2003
Rhaetian	True reef	1	Corals, alage	Gurumugl, Papua New Guinea	Kiessling et al., 2003
Rhaetian	True reef	1	Corals, alage	Albenza, Southern Alps, Italy	Kiessling et al., 2003
Rhaetian	True reef	1	Calcisponges, corals	Begunjscica, Slovenia	Kiessling et al., 2003
Rhaetian	Reef mound	1	Corals, calcisponges	Rhätikon, Switzerland	Kiessling et al., 2003

Rhaetian	Reef mound	1	Corals, calcisponges	Gruber, Austria	Kiessling et al., 2003
Rhaetian	Reef mound	1	Corals, calcisponges	Ombat Plateau, offshore Australia, Indian Ocean, Indian Ocean	Kiessling et al., 2003
Rhaetian	Reef mound	1	Corals	Zlambachgraben, Austria	Kiessling et al., 2003
Rhaetian	Reef mound	1	Corals	Sarnia Skala, Maly Kopieniec, Carpathians, Poland	Kiessling et al., 2003
Rhaetian	Reef mound	1	Corals	E-Sulawesi, Indonesia	Kiessling et al., 2003
Rhaetian	Reef mound	1	Calcisponges, algae	Waliabad, Iran	Kiessling et al., 2003
Rhaetian	Reef mound	1	Serpulids, worms	Rio Blanco, Spain	Kiessling et al., 2003
Rhaetian	Biostrome	1	Corals	Fischerwiese, Steinplatte, Austria	Kiessling et al., 2003
Rhaetian	Biostrome	1	Corals	Mala Fatra, Slovakia	Kiessling et al., 2003
Rhaetian	Biostrome	1	Corals	Hallstätter Salzberg, Austria	Kiessling et al., 2003
Rhaetian	Biostrome	1	Corals	Val Adrara and others, Lombardia, Italy	Kiessling et al., 2003
Rhaetian	Biostrome	1	Corals, calcisponges	Bobrovcek, W Tatra, Slovakia	Kiessling et al., 2003
Rhaetian	Biostrome	1	Corals, algae	Denti della Vecchia, Lugano, Southern Alps, Italy	Kiessling et al., 2003
			Early Juassic		
Early Hettangian	Biostrome	>12	Microbea	St. Audries Bay and Lavernock Point, United Kingdom	Ibarra et al., 2014
Hettangian	True reef	1	Corals, algae	Partizansk, South Primorye, Russia	Kiessling et al., 2003
Hettangian	True reef	1	Corals, microbes	Apuane Alps, Tuscany, Italy	Kiessling et al., 2003
Hettangian	True reef	1	Corals, microbes	Aubenas, Ardeche, France	Kiessling et al., 2003
Hettangian	True reef	1	Corals	Sutton Stone, Southerndown, South	Kiessling et al., 2003
Hettangian	Biostrome	2	Microbes	Kulla Gunnarstorp, Sweden	Peterffy et al., 2016
Hettangian-	True reef	1	Corals	Jebel Bou Mokhta, Morocco	Kiessling et al., 2003

Sinemurian					
Hettangian-Sinemurian	True reef	1	Corals	Mynhajir Zone, SE Pamir, Tadzhikia, Tajikistan	Kiessling et al., 2003
Hettangian-Sinemurian	True reef	1	Corals	Jebel Bou Mokhta, Morocco	Kiessling et al., 2003
Hettangian-Sinemurian	Biostrome	1	Non-rudist bivalves	Johnson Creek, Yukon, Canada	Kiessling et al., 2003
Hettangian-Sinemurian	Biostrome	1	Non-rudist bivalves	Johnson Creek, Yukon, Canada	Kiessling et al., 2003
Sinemurian	True reef	1	Corals	Telkwa Range, British Columbia, United States	Kiessling et al., 2003
Sinemurian	Reef mound	>1	Microbes, sponges	jebelBou Dahar, High Atlas, Morocco	Porta et al., 2013
Sinemurian	Biostrome	1	Corals	Socosani, Arequipa, Peru	Kiessling et al., 2003
Sinemurian	Biostrome	1	Corals	Djarty-Rabat, SE Pamir, Tadzhikia, Tajikistan	Kiessling et al., 2003
Sinemurian	Biostrome	1	Corals	Jebel Bou Iblane, Morocco	Kiessling et al., 2003
Sinemurian	Biostrome	1	Corals, algae	Bou Arnhous, Morocco	Kiessling et al., 2003
Sinemurian	Biostrome	1	Non-rudist bivalves	Zardeh Kuh, Iran	Kiessling et al., 2003
Sinemurian	Biostrome	1	Non-rudist bivalves, corals	Val Stgira, Switzerland	Kiessling et al., 2003
Sinemurian	Biostrome	1	Microbes	Adnet, Austria	Kiessling et al., 2003
Late Cretaceous					
Early Maastrichtian	True reef	1	Non-rudist bivalves	Garzan Field, Turkey	Kiessling et al., 2003
Early	True reef	1	Rudists	Monte San Angelo, Gargano, Italy	Kiessling et al., 2003

Maastrichtian				Cuatretonda, Valencia, Spain	
Early Maastrichtian	Reef mound	1	Rudists, corals	Zlambachgraben, Austria	Kiessling et al., 2003
Early Maastrichtian	Biostrome	1	Rudists, corals	Cerro Cebollin, Bolsón, Costa Rica	Kiessling et al., 2003
Middle Maastrichtian	True reef	1	Rudists, corals	Aroyo de la Atarjea, San Luis Potosi, Mexico	Kiessling et al., 2003
Middle Maastrichtian	Biostrome	1	Rudists	Antikyra, Greece	Kiessling et al., 2003
Middle-Late Maastrichtian	True reef	1	Corals, Rudists	Aroyo de la Atarjea, San Luis Potosi, Mexico	Kiessling et al., 2003
Late Maastrichtian	True reef	1	Corals	Jebel Rawdah, U.A.E., United Arab Emirates	Kiessling et al., 2003
Late Maastrichtian	True reef	1	Corals	Jebel Huwayyah, U.A.E., United Arab Emirates	Kiessling et al., 2003
Late Maastrichtian	True reef	1	Corals, algae	Jumilla-Yecla Altiplano, SE Spain, Spain	Kiessling et al., 2003
Late Maastrichtian	Reef mound	1	Non-rudist bivalves	Estancia el Carmen, Chubut, Argentina	Kiessling et al., 2003
Late Maastrichtian	Reef mound	1	Rudists, algae	Aqra, Iraq	Kiessling et al., 2003
Late Maastrichtian	Biostrome	1	Non-rudist bivalves	Quebrada de la Toma, Chubut, Argentina	Kiessling et al., 2003
Late Maastrichtian	Biostrome	1	Rudists, corals	Barranc del Racó, Bocairent, Spain	Kiessling et al., 2003
Maastrichtian-D	True reef	1	Corals, foraminifers	Tinja, Bosnia, Bosnia and	Kiessling et al., 2003

anian				Herzegovina	
Maastrichtian-Danian	True reef	1	Corals, algae	Mt. Medvednica, Croatia	Kiessling et al., 2003
Maastrichtian-Danian	Reef mound	1	Bryozoans	Stevns Klint, Denmark	Kiessling et al., 2003
Maastrichtian	True reef	1	Rudists	Central Inlier, Jamaica	Kiessling et al., 2003
Maastrichtian	True reef	1	Rudists, corals	La Popa, Mexico	Kiessling et al., 2003
Maastrichtian	True reef	1	Corals, microbes	Tisjé, Somalia	Kiessling et al., 2003
Maastrichtian	Reef mound	1	Rudists	Green Island Inlier, Jamaica	Kiessling et al., 2003
Maastrichtian	Reef mound	1	Rudists, corals	Sivertan Hill, Turkey	Kiessling et al., 2003
Maastrichtian	Reef mound	1	Rudists, corals	Portopalo, Pachino, Sicily, Italy	Kiessling et al., 2003
Maastrichtian	Reef mound	1	Rudists, corals	Vrbovac, Bacevica, Serbia, Yugoslavia	Kiessling et al., 2003
Maastrichtian	Reef mound	1	Rudists, corals	Maldon Inlier, Jamaica	Kiessling et al., 2003
Maastrichtian	Reef mound	1	Corals, algae	Vapenkova skala, Rozbehy, Slovakia	Kiessling et al., 2003
Maastrichtian	Biostrome	1	Rudists	Lambs River, Marchmont Inlier, Jamaica	Kiessling et al., 2003
Maastrichtian	Biostrome	1	Rudists	Coamo, Puerto Rico, United States	Kiessling et al., 2003
Maastrichtian	Biostrome	1	Rudists	Fundina, Podgorica, Montenegro, Yugoslavia	Kiessling et al., 2003
Maastrichtian	Biostrome	1	Rudists	Qarn Murrah, Oman	Kiessling et al., 2003
Maastrichtian	Biostrome	1	Rudists	Jebel Huwayyah, Oman	Kiessling et al., 2003
Maastrichtian	Biostrome	1	Non-rudist bivalves	Willow Creek, Drumheller, Alberta, Canada	Kiessling et al., 2003
Maastrichtian	Biostrome	1	Non-rudist bivalves	Logan Co., North Dakota, United	Kiessling et al., 2003

				States	
Maastrichtian	Biostrome	1	Rudists, algae	Vening Meinesz seamounts, Indian Ocean, Indian Ocean	Kiessling et al., 2003
Maastrichtian	Biostrome	1	Rudists, corals	Sirataslar Ridge, Maden, Turkey	Kiessling et al., 2003
Maastrichtian	Biostrome	1	Corals	Maastricht, Netherlands	Kiessling et al., 2003
Paleocene					
Maastrichtian-Paleocene	True reef	1	Corals, foraminifers	Tinja, Bosnia, Bosnia and Herzegovina	Kiessling et al., 2003
Early Danian	True reef	1	Corals, algae	Biormaiza-2, Pyrenees, Spain	Kiessling et al., 2003
Early Danian	True reef	1	Corals, algae	Puelén, La Pampa, Argentina	Kiessling et al., 2003
Middle Danian	Reef mound	1	Bryozoans, corals	Limhamn, Sweden	Kiessling et al., 2003
Middle Danian	Reef mound	1	Corals, bryozoans	Fakse, Denmark	Kiessling et al., 2003
Late Danian	True reef	1	Corals, algae	Dolenja Vas, Slovenia	Kiessling et al., 2003
Late Danian	True reef	1	Corals, algae	Dax, Aquitaine, France	Kiessling et al., 2003
Late Danian	True reef	1	Corals, algae	Biormaiza and Lizarraga, Pyrenees, Spain	Kiessling et al., 2003
Late Danian	True reef	1	Corals	NE Tarbes, Aquitaine, France	Kiessling et al., 2003
Danian	True reef	1	Corals, algae	Abu Tartur SE, Kharga Oasis, Egypt	Kiessling et al., 2003
Danian	True reef	1	Corals, algae	Kambühel, Austria	Kiessling et al., 2003
Danian	True reef	1	Corals, algae	Zilina II, Slovakia	Kiessling et al., 2003
Danian	True reef	1	Corals, algae	Dündarli, Kayseri, Turkey	Kiessling et al., 2003
Danian	True reef	1	Corals, algae	Vigny, Paris Basin, France	Kiessling et al., 2003
Danian	True reef	1	Corals, algae	Schafkogel, Mariazell, Austria	Kiessling et al., 2003
Danian	True reef	1	Corals, algae	Wörschach, Austria	Kiessling et al., 2003
Danian	True reef	1	Corals, bryozoans	Sendurai, Tamil Nadu, India	Kiessling et al., 2003

Danian	True reef	1	Algae, corals	Amazonas mouth XIII, Brazil	Kiessling et al., 2003
Danian	Reef mound	1	Algae, corals	Maiella, Italy	Kiessling et al., 2003
Danian	Reef mound	1	Algae, bryozoans	Myjava, Slovakia	Kiessling et al., 2003
Danian	Reef mound	1	Bryozoans	Karlby Klint, Denmark	Kiessling et al., 2003
Danian	Reef mound	1	Bryozoans	Hanstholm, Denmark	Kiessling et al., 2003
Danian	Reef mound	1	Corals	Libyan Desert, Egypt	Kiessling et al., 2003
Danian	Reef mound	1	Corals, algae	Corozal, Puerto Rico, United States	Kiessling et al., 2003
Danian	Reef mound	1	Corals, bryozoans	Nûgssuaq, Greenland, Denmark	Kiessling et al., 2003

REFERENCES CITED IN SUPPLEMENTARY TABLE 1

Antoshkina, A.I. Organic buildups and reefs on the Palaeozoic carbonate platform margin, Pechora Urals, Russia. *Sed. Geol.*, **118**, 87-211 (1998).

Antoshkina, A.I. Palaeoenvironmental implications of *Palaeomicrocodium* in Upper Devonian microbial mounds of the Chernyshev Swell, Timan-northern Ural Region. *Facies* **52**, 611-625 (2006).

Aretz, M. & Chevalier, E. After the collapse of stromatoporid-coral reefs—the Famennian and Dinantian reefs of Belgium: much more than Waulsortian mounds in Palaeozoic Reefs and Bioaccumulations: Climatic and Evolutionary Controls (ed. Álvaro, J.J.), *Geological Society, London, Special Publications* **275**, 163-188 (2007).

Berkowski, B. Famennian Rugosa and Heterocorallia from Southern Poland. *Palaeontologia Pol* **61**, 3-88 (2002).

Bhargava, O.N. Carbonate build-ups in the Himalaya: their age, microfacies and palaeoenvironment: *J. Palaeont. Soc. India* **42**, 19-33 (1997).

Brayard, A. *et al*. Transient metazoan reefs in the aftermath of the end-Permian mass extinction. *Nature Geosci*. **4**, 693-697 (2011).

Burg, J.S. Microbially induced sedimentary structures in the Carboniferous Horton Bluff Formation near Hantsport, Nova Scotia. 1-71 (Dalhousie University, Nova Scotia, 2013).

Chow, N.& George, A.D., 2004, Tepee-shaped agglutinated microbialites: an example from a Famennian carbonate platform on the Lennard Shelf, northern Canning Basin, Western Australia. *Sedimentology* **51**, 253-265 (2004).

Draganits, E., Mawson, R., Talent, J.A.& Krystyn, L. Lithostratigraphy, conodont biostratigraphy and depositional environment of the Middle Devonian (Givetian) to Early Carboniferous (Tournaisian) Lipak Formation in the Pin Valley of Spiti (NW India). *R.Ital. Paleont Strat* **108**, 7-35 (2002).

Gallacher, A.F. Microbially mediated sedimentary structures and stromatolites in the Mississippian strata of the Horton Bluff Formation, Nova Scotia. 1-93 (Dalhousie University, Nova Scotia, 2010).

George, A.D.& Chow, N. The depositional record of the Frasnian/Famennian boundary interval in a fore-reef succession, Canning Basin, Western Australia. *Palaeogeogr. Palaeoclimatol. Palaeoecol* 181 (2002).

George, A.D., Playford, P.E., Powell, C.M.& Tornatora, P.M. Lithofacies and sequence development on an Upper Devonian mixed carbonate-siliciclastic fore-reef slope, Canning Basin, Western Australia. *Sedimentology* **44**, 843-867 (1997).

Gutschick, R.C.& Perry, T.G. Sappington (Kinderhookian) sponges and their environment. *J. Pal*. **33**, 977-985 (1959).

Halim-Dihardja, M.K.& Mountjoy, E.W. A stromatoporoid patch reef in the Upper Devonian Wabamun Group, Normandville Field, north-central Alberta. *Can. Soc. Petro Geol Memoir* **13**, 448-453 (1989).

Herbig, H.-G.& Weber, H.M. Facies and stromatoporoid biostromes in the Strunian (latest Devonian) of the Aachen region, Germany. Gött. Arb. Geol. Paläont. **2**, 359-364 (1996).

Ibarra, Y., Corsetti, F. A., Greene, S. E. & Bottjer, D. J. Microfacies of the Cotham marble: a tubestone carbonate microbialite from the Upper Triassic, southwestern U.K. *Palaios* **29**, 1-15 (2014).

Hou, H.F., Zhou, H.L., Liu, J.B. Microbial sediments occurring after the end-Dvonian extinction event on the Hunan platform. *Acta Geol. Sin.* **85**, 145-156 (2011).

Kiessling, W. *et al*. The Paleoreefs Project. www.paleo-reefs.pal.uni-erlangen.de (2003).

Mata, S. A. & Bottjer, D. J. Microbes and mass extinctions: paleoenvironmental distribution of microbialites during times of biotic crisis. *Geobiology* **10**, 3-24 (2012).

Milhau, B. *et al*. Comparative faunal content of Strunian (Devonian) between Etaoucun (Guilin, Guangxi, South China) and the stratotype area (Etroeungt, Avesnois, north of France): Proc. 30th Intern. Geol. Cong. **12**, 79-94. (1997)

Morrow, J., Harries, P.J.& Krivanek, J.G. Reef recovery following the Frasnian-Famennian (Late Devonian) mass extinction: evidence from the Dugway Range, west-central Utah. *Palaios* **26**, 607-622 (2011).

Mundy, D.J.C. Microbialite–sponge–bryozoan–coral framestones in Lower Carboniferous (Late Viséan) buildups of Northern England (UK). *CSPG, Mem.* **17**, 713–729 (1994).

Nguyen, H.H.& Mistiaen, B. Uppermost Famennian stromatoporoids of north central Viet Nam. *Jour. Geology* **11**, 57-75 (1998).

Peterffy, O., Calner, M., Vajda, V. Early Jurassic microbial mats—A potential response to reduced biotic activity in the aftermath of the end-Triassic mass extinction event. *Palaeogeogr*. *Palaeoclimatol*. *Palaeoecol*. doi.org/10.1016/j.palaeo.2015.12.024 (2016).

Peterhänsel, A.& Pratt, B.R. The Famennian (Upper Devonian) Palliser Platform of western Canada—architecture and depositional dynamics of a postextinction epeiric giant, *in* Pratt, B.R. & Holmden, C., eds., Dynamics of Epeiric Seas. *Geol. Soc. Can. Spec. Paper* **48**, 247-281. (2008).

Porta, G.D., Antonio, M.T., Kenter, J. & Verwer, K. Lower Jurassic microbial and skeletal carbonate factories and platform geometry (Djebel Bou Dahar, High Atlas, Morocco). *SEPM Spec. Publ.* **105**, 237-263 (2013).

Pratt, B.R. & Peterhansel, A. Upper Devonian (Famennian) Reefs, Palliser (Wabamun) Formation: Rapid Post-Extinction Recovery in the Canadian Rocky Mountains. Sedimentary Events, Hydrocarbon Systems-CSPG-SEPM Joint Convention: Program Abstracts, 229-229 (1997).

Rakociński, M. & Racki, G. Microbialites in the shallow-water marine environments of the Holy Cross Mountains (Poland) in the aftermath of the Frasnian-Famennian biotic crisis. *Global Planet. Change* **136**, 30-40 (2016).

Sheehan, P. M. & Harris, M. T. Microbialite resurgence after the Late Ordovician extinction. *Nature* **430**, 75-78 (2004).

Shen, J.W.& Webb, G.E. Famennian (Upper Devonian) calcimicrobial (Renalcis) reef at Miaomen, Guilin, Guangxi, South China. *Palaeogeogr. Palaeoclimatol. Palaeoecol*. **204**, 373-394 (2004a).

Shen, J.W.& Webb, G.E., Famennian (Upper Devonian) stromatolite reefs at Shatang, Guilin, Guangxi, South China. *Sed. Geo*l. **170**, 63-84, (2004b).

Shen, J.W.& Zhang, S.L. A Frasnian coral-bafflestone reef Houshan, Guilin, South China. *Facies* **37**, 85-98 (1997).

Shen, J.W., Zhao, N., Mao, Y,J., Wang, Y. & Jin, Y.B. Late Devonian reefs and microbialite in Maoying, Ziyun County of southern Guizhou, South China—Implications for changes in paleoenvironment. *Palaeogeogr. Palaeoclimatol. Palaeoecol*. (2016),

http://dx.doi.org/10.1016/j.palaeo.2016.07.005.

Simakov, K.V. *et al*. Upper Famennian and Tournaisian deposits of the Omolon Region. *Ann. Soc. Géol. Bel* **106**, 335-399 (1983).

Stearn, C.W. Stromatoporoids from the Famennian (Devonian) Wabamun formation, Normandville oilfield, north-central Alberta, Canada. *J. Pal.* **62**, 411-419 (1998).

Stephens, N.P. & Sumner, D.Y. Famennian microbial reef facies, Napier and Oscar Ranges, Canning Basin, western Australia. *Sedimentology* **50**, 1283-1302 (2002).

Tong, Z.S. Characteristics of lithofacies and palaeogeography of the early Carboniferous Qianheishan age in eastern part of northern Qilian Mt. *J Strat* **20**, 190-195. (1996).

Ulmishek, G.F. Upper Devonian-Tournaisian Facies and Oil Resources of the Russian Craton's Eastern Margin, *in* McMillan, N. J., et al., eds., Devonian of the World, Volume I: Regional syntheses. *Can. Soc. Petro Geol Memoir* **14**, 527-549. (1998).

Webb, G.E. Earliest known Carboniferous shallow-water reefs, Gudman Formation (Tn1b), Queensland, Australia: Implications for Late Devonian reef collapse and recovery: *Geology* **26**, 951-954 (1998).

Webb, G.E. Famennian mud-mounds in the proximal fore-reef slope, Canning Basin, Western Australia: *Sed. Geol.* **145**, 295-315 (2001).

Webb, G.E. Quantitative Analysis and Paleoecology of Earliest Mississippian Microbial Reefs, Gudman Formation, Queensland, Australia: Not Just Post-Disaster Phenomena. *J. Sed. Res.* **75**, 877-896 (2005).

Webby, B. D. Patterns of Ordovician reef development. *SEPM Spec. Publ.* **72**, 129-179 (2002).

Whalen, M.T., Day, J., Eberli, G.P. & Homewood, P.W. Microbial carbonates as indicators of environmental change and biotic crises in carbonate systems: examples from the Late Devonian, Alberta basin, Canada. *Palaeogeogr. Palaeoclimatol. Palaeoecol.* **181**, 127-151 (2002).

Wood, R. Novel paleoecology of a post extinction reef: Famennian (Late Devonian) of the Canning basin, northwestern Australia: *Geology* **28**, 987-990 (2000).

Yao, L. et al. Middle Viséan (Mississippian) coral biostrome in central Guizhou, southwestern China and its palaeoclimatological implications. *Palaeogeogr. Palaeoclimatol. Palaeoecol.* **448**, 179-194 (2016).
